# BACE2‐Induced Aberrant Lymphatic Network Aggravates the Local Inflammation in Arteriovenous Fistulas With Hyperphosphatemia

**DOI:** 10.1002/advs.202509632

**Published:** 2025-08-30

**Authors:** Kai Chen, Ziyin Guan, Hanwei Jin, Muladili Aihemaiti, Rong Mou, Mengting Yu, Yanhua Hu, Liujun Jiang, Xuliang Wang, Hao Yang, Ting Chen, Yao Lu, Hongkun Zhang, Qingbo Xu, Weidong Li

**Affiliations:** ^1^ Departments of Cardiology the First Affiliated Hospital Zhejiang University School of Medicine Hangzhou 310003 China; ^2^ Department of Vascular Surgery the First Affiliated Hospital Zhejiang University School of Medicine Hangzhou 310003 China; ^3^ Department of Cardiovascular Surgery the First Affiliated Hospital Zhejiang University School of Medicine Hangzhou 310003 China; ^4^ Department of Nephrology the First Affiliated Hospital Zhejiang University School of Medicine Hangzhou 310003 China; ^5^ Department of Cardiology and the Center of Clinical Pharmacology the Third Xiangya Hospital Central South University Changsha 410013 China

**Keywords:** arteriovenous fistulas, fibro‐progenitor, hyperphosphatemia, lymphatic network, VEGFR3 cleavage

## Abstract

As a widely used vascular access for hemodialysis patients, arteriovenous fistula (AVF) still faces high failure rates, in which local inflammatory response is an essential factor. In animal studies, chronic kidney disease (CKD) has been reported to aggravate local inflammation in AVFs, but the mechanisms are controversial. Here, spatial transcriptomics and single‐cell RNA sequencing are used to explore the cellular changes during AVF remodeling in human and mouse. Lymphatic network, facilitated by a group of *Pi16* ^+^ *Vegfc* ^+^ fibro‐progenitors, is revealed as an overlooked efflux tunnel to avoid extensive inflammatory retention in AVFs. In C57BL/6 mice with 5/6 nephrectomy, the elevated phosphorus impaired AVF lymphatic network by increasing soluble VEGFR3 to blunt vascular endothelial growth factor (VEGF)‐C sensitivity in lymphatic endothelial cells (LECs), for which the increased SP1/BACE2/VEGFR3 cleavage is the underlying mechanism. By creating LEC specific BACE2 knockout mice or applying BACE2 inhibitors in the anastomotic area, the lymphatic network in 5/6‐nephrectomy mouse AVFs is normalized, which alleviated local inflammation and neointima formation. Considering that the hyperphosphatemia is a common metabolic disorder for pre‐dialysis CKD patients, this study provides a novel immunoregulation strategy for AVFs under CKD condition, as suppressing BACE2‐mediated VEGFR3 cleavage to recover a functionally competent lymphatic network is a potential target.

## Graphical abstract

Vascular endothelial growth factor (VEGF)‐C secreted by Pi16^+^ fibro‐progenitors lays the foundation of lymphatic network in mouse arteriovenous fistulas (AVFs), whereas the elevated phosphate (Pi) in 5/6‐nephrectomy (Nx) mouse activates p‐ERK/p‐SP1/BACE2 pathway to generate additional soluble VEGFR‐3, which causes VEGF‐C unresponsiveness and defective lymphatic network with aggravated local inflammation in AVFs under hyperphosphatemia condition.

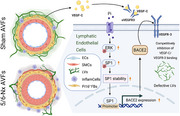

## 1 Introduction

Ever since arteriovenous fistula (AVF) was first created over 50 years ago^[^ ^1^ ^]^ this vascular access has been extensively used for end‐stage renal disease (ESRD) patients requiring hemodialysis. However, only 60% of AVFs could keep unobstructed after 1 year post surgery,^[^ ^2^ ^]^ and 50% of AVFs require interventions to maintain use or treat complications.^[^ ^3^ ^]^ Previously, clinical studies have suggested C‐reactive protein^[^ ^4^, ^5^ ^]^ and other inflammatory biomarkers^[^ ^6^ ^]^ as a prognostic risk factor for AVF failure. Several animal studies^[^ ^7^, ^8^ ^]^ have also indicated that local inflammation negatively influenced AVF remodeling, especially under chronic kidney diseases (CKD) condition.^[^ ^9^ ^]^ For the mechanisms, including macrophage migration inhibitory factor,^[^ ^10^ ^]^ vascular endothelial growth factors (VEGFs),^[^ ^11^ ^]^ and inflammatory cytokines (TNF‐*α* ^[^ ^12^ ^]^/TGF‐*β* ^[^ ^13^ ^]^) have been referred. Meanwhile, as a common metabolic disorder for pre‐dialysis CKD patients, though hyperphosphatemia have been connected with AVF dysfunction,^[^ ^14^, ^15^ ^]^ there still lacks of reasonable explanation and pharmacological targets, especially from inflammatory perspective.

Recently, lymphatic network has been reported to impact vascular diseases in multiple ways. In atherosclerosis^[^ ^16^ ^]^ or aortic aneurysms,^[^ ^17^ ^]^ efficient lymphatic drainage is beneficial for the lesion regression due to the improved clearance of inflammatory cells. On the contrary, lymphatic network is inclined to aggravate transplant arteriosclerosis by assisting tertiary lymphoid organs formation to maintain adaptive immunity.^[^ ^18^, ^19^ ^]^ Such controversy is attributed to the discrepant immune environment between different vascular diseases. On the other hand, while VEGFs dominantly regulate lymphangiogenesis in vascular diseases,^[^ ^19^, ^20^ ^]^ some novel orchestrations of lymphatic vasculature, including oxidative phosphorylation,^[^ ^21^ ^]^ fatty acid/glucose metabolisms,^[^ ^22^, ^23^ ^]^ and VEGFR3 cleavage pathway,^[^ ^24^ ^]^ were also uncovered. However, it is still unknown whether and how lymphatic network would affect AVF remodeling, especially under CKD condition.

In this study, we depicted a cellular landscape of AVFs by spatial transcriptomics and single‐cell RNA sequencing (scRNA‐seq). Pi16^+^ fibro‐progenitors facilitated lymphatic network was revealed as an overlooked structure to prevent excessive inflammatory reaction in AVFs. We found that elevated phosphate in 5/6‐nephrectomy mice impaired AVF lymphatic network by reducing VEGF‐C sensitivity in lymphatic endothelial cells (LECs), in which upregulated SP1/BACE2 was the molecular mechanism. By applying BACE2 inhibitors in the anastomosis area, we rectified the defective lymphatic network and alleviated the local inflammation in mouse AVFs with 5/6 nephrectomy, implicating a therapeutic immunoregulation strategy for AVFs with hyperphosphatemia.

## 2 Results

### 2.1 Spatial Transcriptomics Reveals the Fibrogenesis‐Facilitated Lymphangiogenesis in Human AVFs

**Figure 1:**
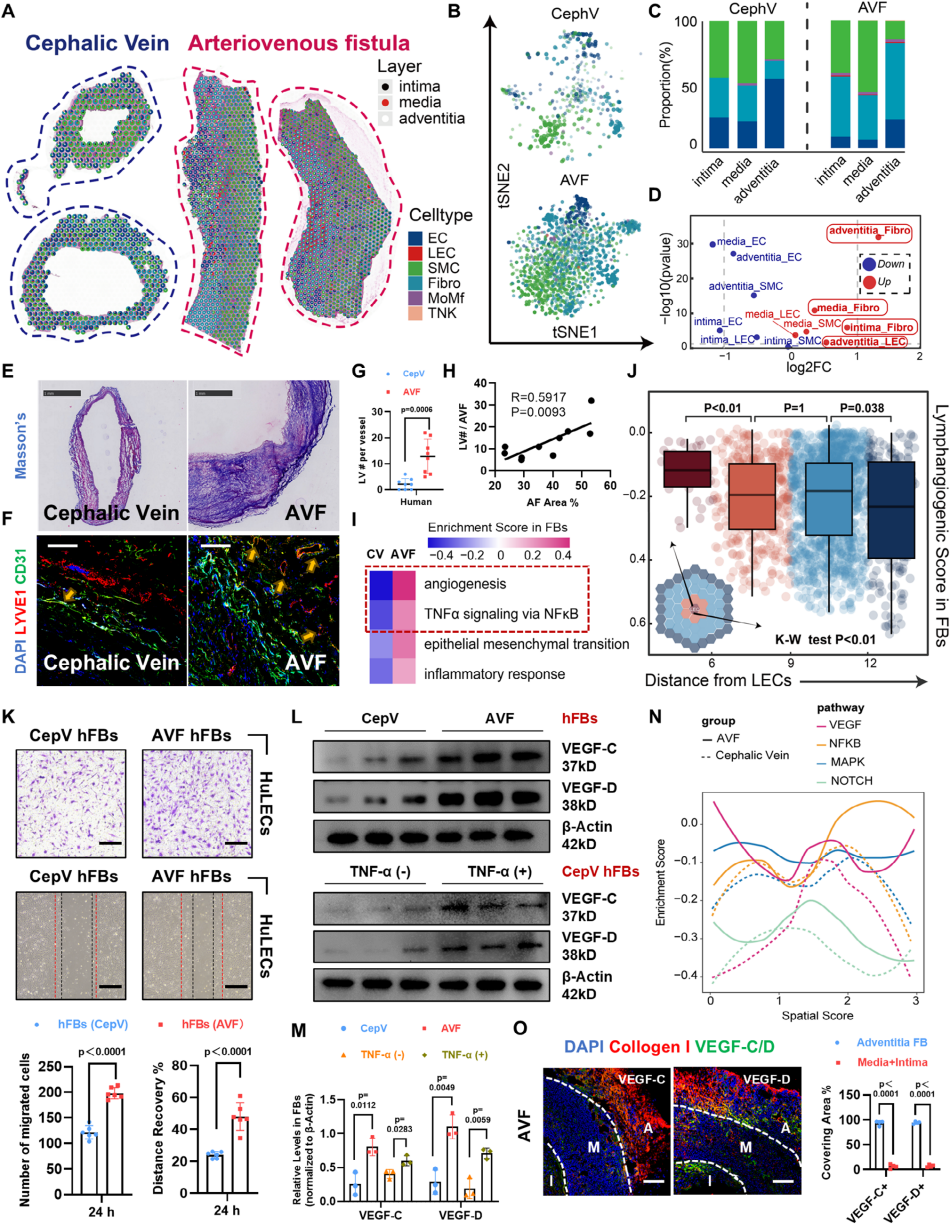
The connection between fibrosis and lymphangiogenesis in human arteriovenous fistulas (AVFs). A) Scatter pie plot displaying the cell‐type proportions by “cell2location” estimation at each spot, with dot plot (inner side) showing vascular layers of each spot (cephalic veins, *n* = 186 or 313; venous segments of AVFs, *n* = 892 or 933). B) By using harmony method to respectively merge cephalic vein or AVF spots, t‐distributed stochastic neighbor embedding (tSNE) plot showing the spot clusters colored by cell types. C,D) Bar plot showing proportional distributions of different cell types in three layers (C), with volcano plot showing the statistical significances (D). E) Representative Masson's staining for human cephalic vein and AVF sections. F,G) Representative immunostaining for LYVE1 (red) and CD31 (green) in human cephalic vein and AVF sections (F), with comparisons of lymphatic vessel (LV) numbers per vessels (*n* = 10, unpaired two‐tailed Student's *t*‐test) (G); Yellow arrows indicate lymphatic endothelial cells (LECs). H) Linear regression analysis of adventitial fibrosis (AF) area with LV numbers in human AVFs (*n* = 10, linear regression analysis test). I) Heatmap showing top differentiated biological functions between fibroblasts from cephalic veins or AVFs by gene set variation analysis (GSVA). J) Box plot comparing the lymphangiogenic score of fibroblast spots (*n* = 954) according to the distances from lymphatic endothelial cells (LECs); non‐parametric Kruskal–Wallis test was used. K) Scratch and Transwell migration assay of HuLECs transferred with conditional medium from cephalic vein or AVF human fibroblasts; Quantifications shown on the bottom (*n* = 3, unpaired two‐tailed Student's *t*‐test). L,M) Western Blot analyses of VEGF‐C and VEGF‐D in human fibroblasts from cephalic veins or AVFs; or cephalic vein fibroblasts treated with or without TNF‐*α* (100 ng mL^−1^) (L); Quantifications shown on the bottom (*n* = 3, unpaired two‐tailed Student's *t*‐test) (M). N) Line plot showing the relationship between enrichment score of representing pathways with “spatial score”; Calculating method for venous wall classification shown in Figure S1E (Supporting Information). O) Representative immunostaining for COLLAGEN I (red) and VEGF‐C/VEGF‐D (green) in human AVF sections (A, adventitia; M, media; I, intima), with quantification of VEGF‐C/D+ area in different areas (*n* = 4, unpaired two‐tailed Student's *t*‐test). Data are mean ± SD. Scale bars, 1000 µm in (E), 210 µm in (F,O) and 50 µm in (K). *P* values of each comparison were specified in the graph. CepV, cephalic vein; AF, adventitial fibrosis; hFBs, human fibroblasts; and VEGF, vascular endothelial growth factor.

We collected surplus cephalic veins from patients during their first fistulization, and venous segments of AVFs from patients who underwent renal transplant and volunteered to remove AVF structures (**Figures** **1**A and S1A, Supporting Information). After quality control and data normalization (Figure S1B, Supporting Information), we performed spatial transcriptomics analyses in these vessels. According to the representative differentially expressed genes (DEGs), we identified six cell clusters within venous wall, including fibroblasts (DCN/FBLN1), endothelial cells (ECs, *PECAM1*/*VWF*), smooth muscle cells (SMCs, *ACTA2*/*MYH11*), LECs (*MMRN1*/*FLT4*), monocytes/macrophages (Mo/Mf, *C1QA*/*CD14*), and T/NK cells (*GPI*/*DDX24*) (Figure S1C, Supporting Information). Based on the typical cellular components (ECs/*VWF* for intima; SMCs/*ACTA2* for media; fibroblasts/*DCN* for adventitia), we generally divided the venous structures into three layers (Figure S1D, Supporting Information). However, this kind of classification was less accurate because of the inevitably overlapping location of different cells in the same layer. Therefore, we followed the standard definitions in single‐cell spatial transcriptomics of blood vessels,^[^ ^27^ ^]^ and chose to measure the distances to the nearest points at both edges (*D*1, lumen; *D*2, adventitia). By calculating the distance ratios (*D*1/*D*1+*D*2), we classified the venous structures into intima (0–1), media (1–2), and adventitia (2–3) (Figure S1E, Supporting Information). Instead of ECs or SMCs, which have been reported to be the major cellular participants in AVF remodeling,^[^ ^28^ ^]^ the most significant cellular changes of AVFs in our data were the increased fibroblasts and LECs within adventitial layer (Figure 1B–D and Figure S1F, Supporting Information). Using Masson's and immunostaining, we confirmed that the evident adventitial fibrosis (Figure 1E) and LYVE1^+^CD31^+^ lymphangiogenesis (Figure 1F,G) simultaneously occurred post AVF surgery. Unlike two independent process, the severe fibrosis was always accompanied with denser lymphatic network in human AVFs (Figure 1H). Compared with the fibroblasts from cephalic veins, the fibroblasts in AVFs upregulated their angiogenic functions (Figure 1I). Moreover, we compared the lymphangiogenic scores in fibroblasts according to the distances from LECs, and observed that the LEC‐adjacent fibroblasts tended to possess stronger lymphangiogenic functions in AVFs (Figure 1J).

To validate above observation, we cultured the primary fibroblasts from either human cephalic veins or AVFs, and respectively extracted the cellular supernatants to stimulate human LECs (HuLECs) in scratch and Transwell experiments. As the result, fibroblasts from AVF tissues showed an advantageous lymphangiogenic function in related measurements (Figure 1K). Additionally, compared with cephalic veins, we found VEGF‐C/D, the most essential lymphangiogenic factors,^[^ ^29^ ^]^ were more highly expressed in AVF fibroblasts (Figure 1L,M). Considering that the TNF‐*α* signaling, which has been reported to be secreted by ECs and SMCs in AVFs,^[^ ^30^ ^]^ was revealed as the main activating pathway in AVF fibroblasts (Figure 1I), we also used TNF‐*α* to stimulate the fibroblasts from cephalic veins. Resultantly, their VEGF‐C/D expression was consistently upregulated (Figure 1L,M). Similarly, spatial transcriptomics revealed an upregulated VEGF pathway in AVF fibroblasts, especially in the adventitial area (Figure 1N). Using immunostaining, we confirmed that the VEGF‐C/D were majorly expressed by adventitial fibroblasts in human AVFs (Figure 1O). Above information suggests that the lymphangiogenesis and lymphangiogenic fibrosis are two close‐connected but overlooked characteristics in AVF remodeling.

### 2.2 ScRNA‐Seq Confirms the Lymphangiogenic Fibrosis Is a Conservative Characteristic in Human and Mouse AVFs

**Figure 2:**
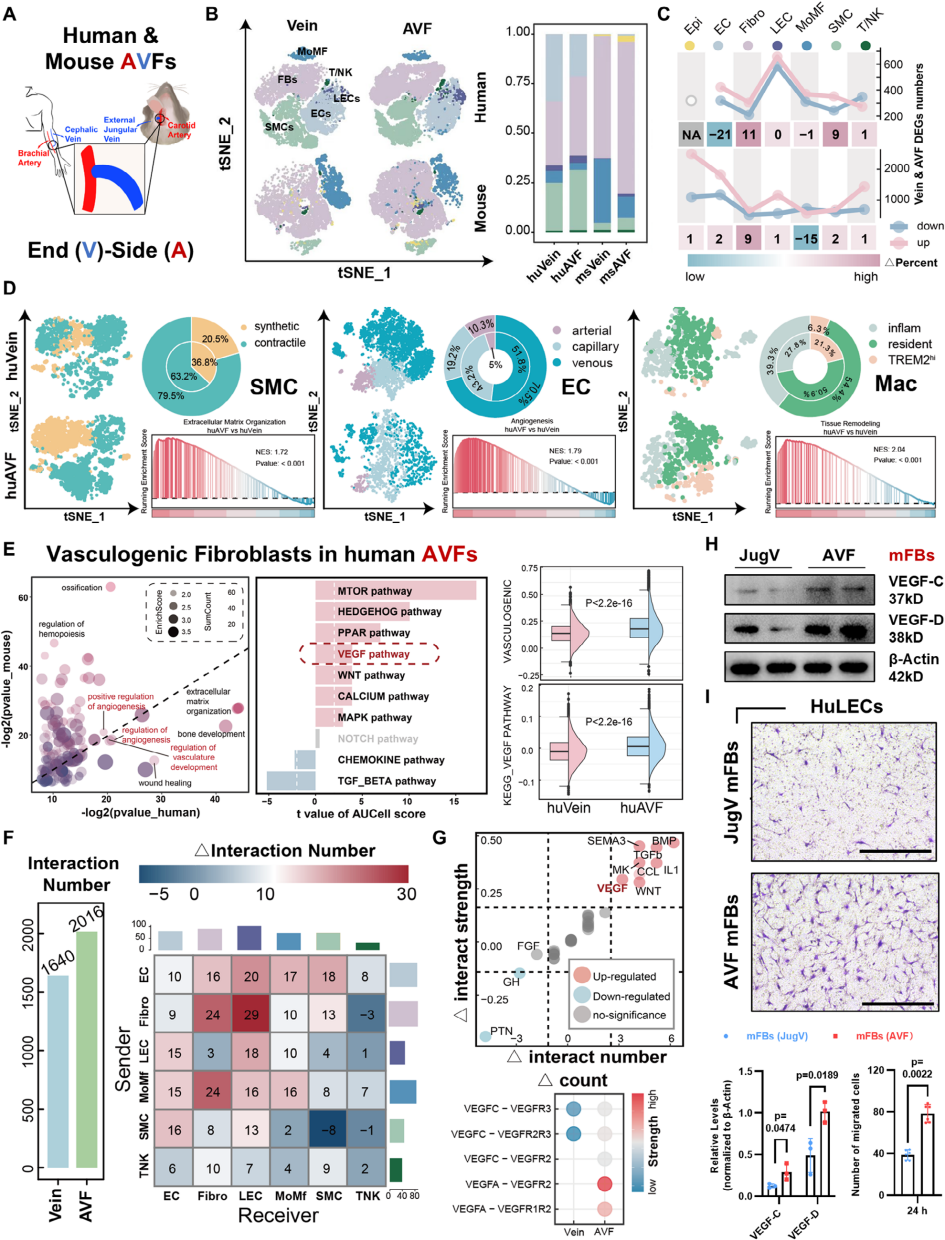
Single‐cell RNA Sequencing (scRNA‐seq) confirmed the lymphangiogenic fibrosis in human and mouse AVFs. A) Schematic diagram illustrating the construction of AVFs in human and mouse. B) tSNE plot showing the identified cell clusters, with bar plot displaying cell‐type proportions (huVein, *n* = 12617; huAVF, *n* = 9277; msVein, *n* = 11922; msAVF *n* = 8385). C) Line plot showing the differentially expressed gene (DEG) numbers (up and down), with heatmap plot displaying the proportional changes of different cell types after fistulation. D) tSNE plot showing the subclusters of smooth muscle cells (SMCs), endothelial cells (ECs), and macrophages (Macs) in human cephalic veins and AVFs, with pie plot displaying the subcluster proportions; Gene set enrichment analysis (GSEA) showing representative enriched functions in each cell type. E) Bubble plot showing the conservatively enriched functions between human and mouse fibroblasts after fistulation, with bar plot emphasizing the VEGF pathway; Box plot comparing the gene set score of “vasculogenesis” or VEGF pathway using “AddModuleScore” between human fibroblasts from cephalic veins and AVFs by unpaired two‐tailed Student's *t*‐test. F) Bar plot displaying total interaction numbers in human cephalic veins or AVFs (left), with heatmap showing the interaction numbers between different cell types (senders or receivers) after fistulation (right). G) Compared with cephalic veins, dot plot showing upregulated or downregulated pathways enriched in the cell–cell interaction from fibroblasts to LECs in AVFs, with bubble plot showing specific receptors and ligands of VEGF‐VEGFR pathways. H) Western Blot analyses of VEGF‐C and VEGF‐D in mouse fibroblasts from jugular veins (JVs) or venous segments of AVFs. I) Transwell migration assay of HuLECs transferred with conditional medium from JV or AVF mouse fibroblasts; Quantifications shown on the bottom (*n* = 3, unpaired two‐tailed Student's *t*‐test). Data are mean ± SD. Scale bars, 100 µm in (I). *P* values of each comparison were specified in the graph. JugV, jugular vein; hu, human; and mFBs, mouse fibroblasts.

In order to prevent the selection bias in spatial transcriptomics to weaken our conclusion, we digested the entire vessels and used scRNA‐seq to compare the entire cellular components between cephalic veins and venous segments of AVFs. In view of that collected tissue were from different patients, for reducing unintentional interference factors, we simultaneously established the mouse cervical AVF models by end‐side anastomosis technique, and again used scRNA‐seq to compare the jugular veins (JVs) and venous segments of AVFs from age‐matched mice (**Figure** **2**A). According to the putative biological identities based on DEGs, we identified seven cell clusters, including fibroblasts (*DCN*/*FBLN1*, human; *Dcn*/*Col1a1*, mouse), ECs (*PECAM1*/*VWF*, human; *Pecam1*/*Aqp1*, mouse), SMCs (*ACTA2*/*MYH11*, human; *Acta2*/*Myh11*, mouse), macrophages/monocytes (*C1QA*/*RGS1*, human; *C1qa*/*Lyz2*, mouse), LECs (*CCL21*/*LYVE1*, human; *Ccl21a*/*Lyve1*, mouse), epithelial (Krt14, mouse), and T/NK cells (*NTS*/*NKG7*, human; *Ets1*/*Il7r*, mouse) (Figure S2A, Supporting Information). Similar with spatial transcriptomics, scRNA‐seq also showed that the increased fibroblasts were one of the representative cellular changes in AVFs (Figure 2B,C). Meanwhile, the distinct subgroup proportions for other cell types, such as SMCs, ECs, or macrophages, indicated a clear vascular remodeling happened post AVF surgery (Figure 2D and Figure S2B–D, Supporting Information). When comparing the general DEGs between cephalic veins and AVFs from human, we found that *CCL21*, a specific gene expressed by LECs, was more highly expressed in human AVFs (Figure S2E, Supporting Information), in accordance with the increased lymphangiogenesis mentioned above (Figure 1F,G). Besides, the upregulated and downregulated DEGs in human LECs had the largest number among all cell types (Figure 2C), indicating that severe genetic changes have occurred in AVF lymphatic network. In both mouse and human, compared with the fibroblasts from control veins, fibroblasts in AVFs were again enriched with higher scores of vasculogenic function and VEGF pathways (Figure 2E and Figure S2F, Supporting Information). Using cell chat analyses, we found the cell–cell interactions between fibroblasts (sender) and LECs (receiver) were the strongest among all mutual signals in AVF cells (Figure 2F and Figure S2G, Supporting Information), in which the acknowledged lymphangiogenic VEGF/VEGFR pathway was one of the represented ligand–receptor pairings (Figure 2G). More directly, the lymphangiogenic property was revealed as the most conservatively upregulated functions of AVF fibroblasts across species (Figure S2H, Supporting Information). In order to prove such comparability, we cultured the primary mouse fibroblasts from either JVs or AVFs, and harvested respective cellular supernatant to stimulate HuLECs in vitro. Expectedly, we confirmed that the mouse AVF fibroblasts had higher VEGF‐C/D expression (Figure 2H,I), which led to a stronger pro‐migration ability in HuLEC Transwell experiments (Figure 2I). These results reflect that the lymphangiogenic fibrosis is a highly conservative characteristic in AVF remodeling.

### 2.3 VEGF‐C/D Dependent Lymphatic Network Is an Overlooked Factor Alleviating Inflammatory Burden in Mouse AVFs

**Figure 3:**
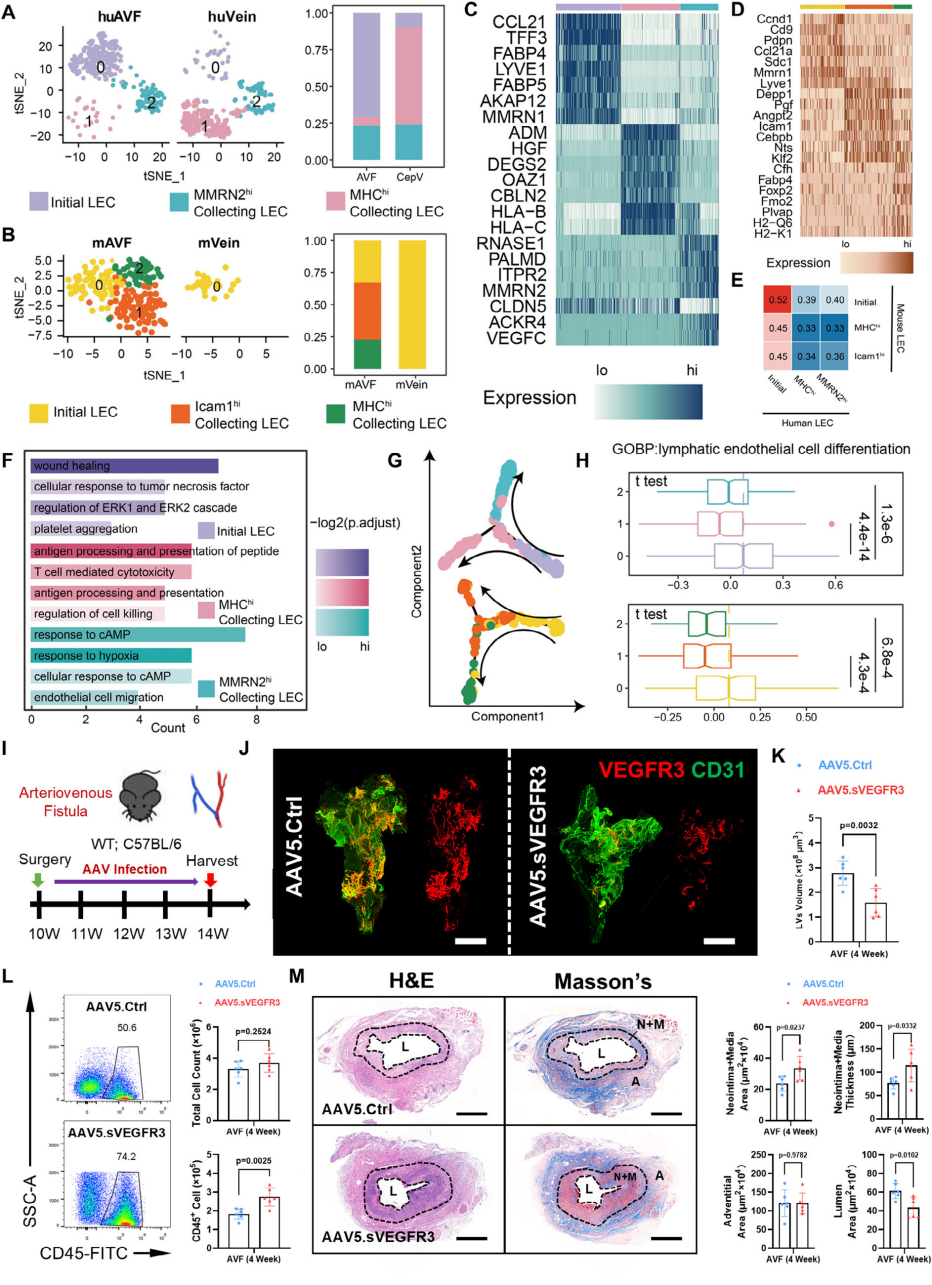
The molecular and functional characteristics of lymphatic network in AVFs. A,B) tSNE plot showing human (A) and mouse (B) LEC subclusters identified by unbiased clustering, with bar plot displaying the discrepant proportions in control veins and venous segments of AVFs. C,D) Heatmap plot showing the expression of top DEGs in each human (C) or mouse (D) LEC subcluster. E) Heatmap plot showing the Pearson correlation score among three LEC subclusters between human and mouse. F) Bar plot showing top differentiated functions enriched in each human LEC subcluster. G,H) Pseudo‐time trajectory of LECs with DDRTree algorithm for dimension reduction in human (top) and mouse (bottom) (G), with box plot showing the gene set score of “lymphatic endothelial cell differentiation” (H). I) Schematic diagram illustrating the adeno‐associated virus (AAV) infection strategy, as AVFs were locally incubated with a hydrogel carrying AAV with noncoding empty vector (Control) or soluble vascular endothelial growth factor receptor 3 (sVEGFR3) vector; Ctrl, control. J,K) Representative whole‐mount staining for VEGFR3 (red) and CD31 (green) in AVFs from WT mice, with AAV.Ctrl (Control) or AAV.sVEGFR3 infection (J) and corresponding quantifications of VEGFR3^+^ LV volume (*n* = 6, unpaired two‐tailed Student's *t*‐test) (K). L) Flow cytometric of CD45^+^ proportions with quantifications of total and CD45^+^ cell counts (*n* = 6, unpaired two‐tailed Student's *t*‐test) in AAV.Ctrl‐ or AAV.sVEGFR3‐infected 4‐week AVFs from WT mice. M) Representative H&E or Masson's staining of AAV.Ctrl‐ or AAV.sVEGFR3‐infected 4‐week AVFs from WT mice, with quantifications of lumen/neointima+media/adventitia areas or “neointima+media” thickness (n = 6, unpaired two‐tailed Student's t‐test). Data are mean ± SD. Scale bars, 1000 µm in (J) and 500 µm in (L). *P* values of each comparison were specified in the graph. N+M, neointima+media.

Although lymphatic network has been reported to impact various vascular diseases,^[^ ^16^, ^19^ ^]^ it is rarely mentioned in AVF remodeling. Using t‐distributed stochastic neighbor embedding (t‐SNE) to visualize the focused clustering of LECs in human and mouse AVFs, some commonalities were first revealed. Basically, both human or mouse AVF LECs could be classified into two categories, initial and collecting LECs (**Figure** **3**A,B). For initial LECs, the DEGs were relatively similar across species, with higher expression of *Lyve1*, *Ccl21*, and *Mmrn1*, et al. (Figure 3A,B). By contrast, DEGs for collecting LECs were quite different. The collecting LECs in mouse AVFs were present as *Icam1* ^hi^ and *MHC* ^hi^ LECs, while the collecting LECs in human AVFs were *MMRN2* ^hi^ and *MHC* ^hi^ LECs (Figure 3A–D). Using correlation analysis, we confirmed that the initial LECs had higher levels of species similarity (Figure 3E), and the functional enrichment of initial LECs also had more overlaps between mouse and human (Figure 3F and Figure S3A, Supporting Information). Considering the construction of lymphatic network always begins with initial LECs,^[^ ^31^ ^]^ we used pseudo‐time analysis and confirmed that the initial LECs had the highest differentiation potentials in both human and mouse AVFs (Figure 3G,H). Moreover, we used *Lyve1*‐Cre^ER^;R26‐tdTomato(tdT) mice^[^ ^19^ ^]^ to perform the lineage tracing study (Figure S3B, Supporting Information). Since *Lyve1* is generally a typical gene for initial LECs but not collecting LECs,^[^ ^32^ ^]^ we successfully labeled all initial LECs around JVs in *Lyve1*‐Cre^ER^;R26‐tdT mice after tamoxifen (Tam) treatment pre‐surgery (Figure S3B, Supporting Information). Notably, even when the initial LECs only covered less than 50% of entire LECs in mouse AVFs (Figure 3B), nearly 100% of the lymphatic network was labeled with tdT signals in AVFs post‐surgery (Figure S3B, Supporting Information), demonstrating that initial LECs are the orientation of AVF lymphatic network. Meanwhile, we used wholemount staining, and observed that the lymphatic network in AVFs had a special growth patten, as the initial LECs first appeared at the anastomotic area, where AVFs are locally exposed to the largest and fastest gradients of wall shear stress,^[^ ^33^ ^]^ and then gradually spread toward the venous segments (Figure S3C, Supporting Information). However, when using conditional medium from AVF fibroblasts to stimulate HuLECs, we did not observe significant upregulation of collecting LEC markers (Figure S3D, Supporting Information), but only activated cellular chemotaxis (Figure S3E, Supporting Information), indicating that the lymphangiogenic fibroblasts were more responsible for the LEC growth rather than maturation.

Except for the origin and distribution characteristics, we explored the assumed functions of lymphatic network in AVFs. Compared with *LYVE1*, which were simultaneously expressed by both LECs and macrophages, *FLT4*, the gene encoding VEGFR3, could only be detected in LECs from AVFs (Figure S4A, Supporting Information). To validate, we created *Prox1*‐Cre^ER^;R26‐tdT mice, in which tdT^+^ signals perfectly labeled the LECs in multiple organs after Tam treatment (Figure S4B–D, Supporting Information). Using *Prox1*‐Cre^ER^;R26‐tdT mice, we detected that nearly 100% of VEGFR3^+^ cells in mouse AVF were tdT^+^ LECs (Figure S4E, Supporting Information). This disease‐specific characteristic indicates that the VEGFR3^+^ structure is a suitable target for visualizing peri‐AVF lymphatic network. Then, we locally incubated the AVF tissue with a hydrogel carrying a control adeno‐associated virus (AAV) or an AAV overexpressing soluble vascular endothelial growth factor receptor 3 (sVEGFR3), the decoy receptor for VEGF‐C/D,^[^ ^34^ ^]^ to decrease lymphatic vessel density (Figure S4F, Supporting Information). Wholemount immunostaining and three‐dimensional reconstruction were used to visualize the lymphatic network in AVFs. Resultantly, we successfully restrained the lymphatic vessel growth in AAV.sVEGFR3‐treated AVFs (Figure 3I–K). Since lymphatic vessels have been reported to undertake the leukocyte trafficking function in atherosclerosis,^[^ ^35^ ^]^ we used flow cytometry and detected increased CD45^+^ proportions in AAV.sVEGFR3‐treated AVFs (Figure 3L), accompanied with aggravated neointima formation (Figure 3M). These data indicate that the construction of lymphatic network is an overlooked protection mechanism in the inflammation‐induced AVF remodeling.

### 2.4 Pi16^+^ Progenitor‐Like Fibroblasts Play an Essential Role in the VEGF‐C Stimulating AVF Lymphangiogenesis

**Figure 4:**
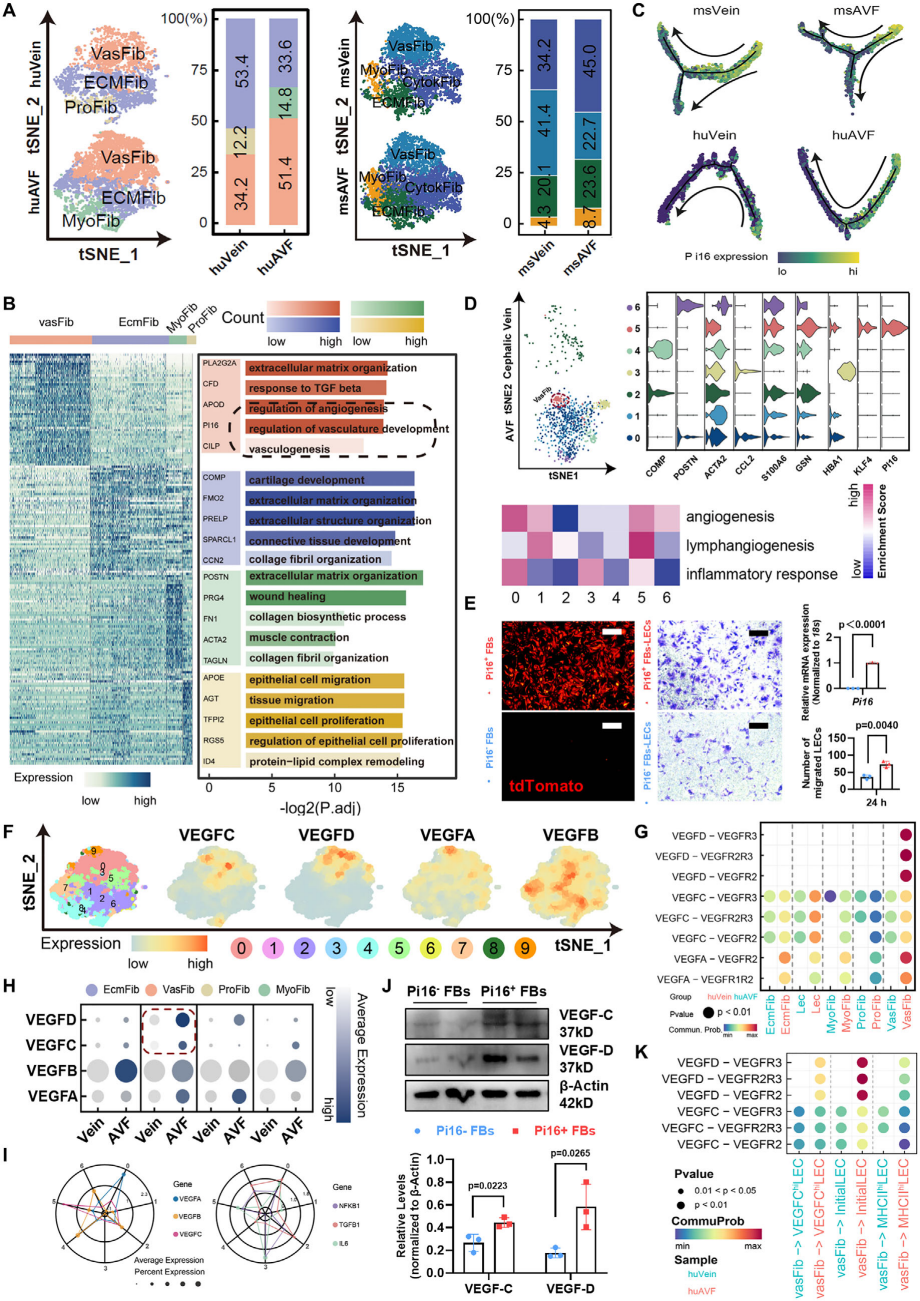
The advantageous lymphangiogenic function of Pi16^+^ fibro‐progenitors in AVFs. A) tSNE plot showing subclusters of human and mouse fibroblasts in control veins and venous segments of AVFs (huVein, *n* = 5480; huAVF, *n* = 3820; msVein, *n* = 5467; msAVF, *n* = 5542), with bar plot displaying proportions. B) Heatmap plot showing the expression of top DEGs in each human fibroblast subcluster, with bar plot displaying corresponding enriched biological functions. C) Pseudo‐time trajectory of human and mouse fibroblasts with DDRTree algorithm for dimension reduction, along with the *Pi16* expression. D) tSNE plot showing the fibroblast subclusters identified by unbiased clustering in human cephalic veins and AVFs (spatial transcriptomics), with violin plot displaying the expression of representative DEGs in each subcluster; Heatmap plot showing the gene set score of “angiogenesis,” “lymphangiogenesis,” and “inflammatory response” for each fibroblast subcluster using “AddModuleScore.” E) Representative immunostaining for tdT (red) in primary tdT/Pi16^+^ or tdT/Pi16^−^ mouse fibroblasts isolated from tamoxifen (Tam)‐treated *Pi16*‐Cre^ER^;R26‐tdT mice, with quantitative PCR analyses of *Pi16*; Transwell migration assay of HuLECs transferred with conditional medium from Pi16^+^ or Pi16^−^ mouse fibroblasts; Quantifications shown on the right (*n* = 3, unpaired two‐tailed Student's *t*‐test). F) Density heatmap showing the expression of VEGFs family in human fibroblasts from AVFs. G) Dot plot comparing the VEGF‐VEGFR communication probability of different fibroblast subtypes to LECs between human cephalic veins and AVFs. H) Bubble plot comparing the expression of VEGFs family in different fibroblast subtypes between human cephalic veins and AVFs. I) Radar plot showing the expression of VEGFs family and inflammation‐associated genes in each fibroblast clusters (spatial transcriptomics). J) Western Blot analyses of VEGF‐C and VEGF‐D in mouse Pi16^+^ or Pi16^−^ fibroblasts; Quantifications shown on the bottom (*n* = 3, unpaired two‐tailed Student's *t*‐test). K) Dot plot comparing the communication probability of vasFib (Pi16^+^ fibroblasts) to different LEC subtypes between human cephalic veins and AVFs. Data are mean ± SD. Scale bars, 50 µm in (E). *P* values of each comparison were specified in the graph. tdT, tdTomato; and vasFib, vasoactive fibroblasts.

Having confirmed that the impairment of lymphatic network could aggravate the local inflammation in mouse AVFs, we continued to elaborate about the lymphangiogenic fibroblasts in detail. Using focused clustering, we identified four fibroblast subclusters in mouse and human AVFs, among which the emerging myofibroblasts was the representative fibrotic change (**Figures** **4**A,B and S5A,B, Supporting Information). Meanwhile, a group of Pi16‐expressing fibroblasts were found to abundantly exist in both human and mouse AVFs (Figure 4A,B and Figure S5A,B, Supporting Information). Previously, Pi16^+^ fibroblasts were regarded as the progenitor‐like fibroblast lineage.^[^ ^36^ ^]^ In AVFs, we also found that the Pi16 expression contrarily reflected the differentiation stages of fibroblasts (Figure 4C and Figure S5C, Supporting Information). Compared with Pi16^−^ fibroblasts, Pi16^+^ fibroblasts had conservatively higher stemness score in both human and mouse AVFs (Figure S5D,E, Supporting Information). Considering their progenitor‐like property, we used *Pi16*‐Cre^ER^;R26‐tdT mice, which could perfectly label Pi16^+^ fibroblasts after Tam treatment.^[^ ^37^ ^]^ Concomitantly, we confirmed that Pi16^+^ fibroblasts could differentiate into myofibroblasts in AVFs, represented by the increased co‐staining of tdT and POSTN (Figure S5G, Supporting Information). Meanwhile, we noticed that the Pi16^+^ fibro‐progenitors were also functionally enriched with vasculature regulation in the scRNA‐seq data of AVFs (Figure 4B and Figure S5A, Supporting Information). The spatial transcriptomics data also confirmed that the Pi16^+^ fibro‐progenitors had the highest lymphangiogenesis scores in human AVFs (Figure 4D). On this account, we sorted Pi16(tdT)^+^ or Pi16(tdT)^−^ fibroblasts from *Pi16*‐Cre^ER^;R26‐tdT veins, and confirmed a stronger lymphangiogenic effect in Pi16^+^ fibroblasts (Figure 4E). Mechanistically, we found that although VEGF family was differentially expressed by fibroblast subclusters (Figure 4F,G), Pi16^+^ fibro‐progenitors always possessed higher VEGF‐C/D expression among AVF fibroblasts (Figure 4H–J). Moreover, such advantageous lymphangiogenic functions was further intensified, as evidenced by the reinforced Pi16^+^ fibroblasts‐LECs interactions post AVF surgery (Figure 4K).

**Figure 5:**
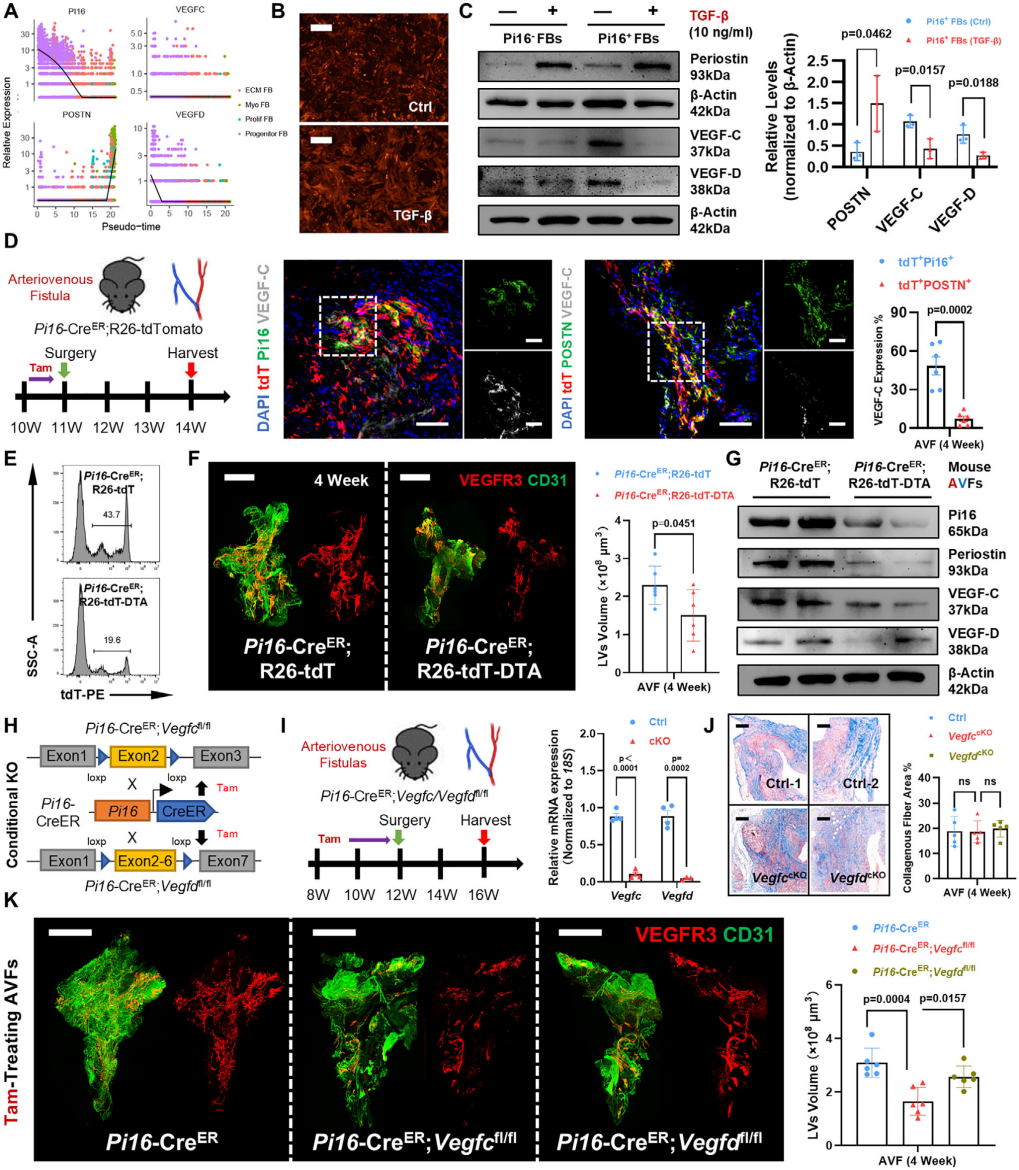
The myo‐activation weaken the lymphangiogenic function of Pi16^+^ fibro‐progenitors in AVFs. A) Curves displaying the relationships between the expression levels of lymphangiogenic genes (*VEGFC*/*VEGFD*) and myo‐activation genes (*POSTN*) or *Pi16* using Pseudo‐time analyses in human fibroblasts from AVFs. B) Representative cellular morphology of mouse tdT/Pi16^+^ fibroblasts, treated with or without TGF‐*β*. C) Western Blot analyses of POSTN, VEGF‐C, and VEGF‐D in Pi16^+^ or Pi16^−^ mouse fibroblasts, treated with or without TGF‐*β*; Quantifications shown on the right (*n* = 3, unpaired two‐tailed Student's *t*‐test). D) Lineage tracing strategy with representative immunostaining for tdT (red), VEGF‐C (gray), and Pi16/POSTN (green) in 4‐week AVF sections from *Pi16*‐Cre^ER^;R26‐tdT mice, with corresponding quantifications of VEGF‐C^+^ proportions in tdT^+^Pi16^+^ or tdT^+^POSTN^+^ cells (*n* = 6, unpaired two‐tailed Student's *t*‐test). E) Representative flow cytometric of tdT^+^ proportions in AVFs from Tam‐treated *Pi16*‐Cre^ER^;R26‐tdT or *Pi16*‐Cre^ER^;R26‐tdT‐DTA mice; Quantifications of tdT^+^ cell counts shown in Figure S5H (Supporting Information). F,G) Representative whole‐mount staining for VEGFR3 (red) and CD31 (green) with corresponding quantifications of lymphatic vessel volume (*n* = 6, unpaired two‐tailed Student's *t*‐test) (F), and Western Blot analyses of Pi16, POSTN, VEGF‐C, and VEGF‐D (G) in AVFs from Tam‐treated *Pi16*‐Cre^ER^;R26‐tdT or *Pi16*‐Cre^ER^;R26‐tdT‐DTA mice. H) Schematic diagram illustrating the construction strategy of *Pi16*‐Cre^ER^;*Vegfc* ^fl/fl^ (*Vegfc* ^cKO^) or *Pi16*‐Cre^ER^;*Vegfd* ^fl/fl^ (*Vegfd* ^cKO^) mice. I) Quantitative PCR analyses of *Vegfc* and *Vegfd* in primary AVF fibroblasts cultured from Tam‐treated control (*Pi16*‐Cre^ER^), *Vegfc* ^cKO^ or *Vegfd* ^cKO^ mice (*n* = 4, unpaired two‐tailed Student's *t*‐test). J) Representative Masson's staining of AVF sections from Tam‐treated control, *Vegfc* ^cKO^ or *Vegfd* ^cKO^ mice, with quantifications of blue color‐represented collagenous fiber covering area (*n* = 6). K) Representative whole‐mount staining for VEGFR3 (red) and CD31 (green) of AVFs from Tam‐treated control, *Vegfc* ^cKO^ or *Vegfd* ^cKO^ mice, with corresponding quantifications of lymphatic vessel volume (*n* = 6, one‐way ANOVA followed with Tukey's post hoc analysis). Data are mean ± SD. Scale bars, 50 µm in (B), 210 µm in (D), 1000 µm in (F,K), and 100 µm in (J). *P* values of each comparison were specified in the graph. *P* > 0.05, ns, nonsignificant.

Meanwhile, we found the myo‐activation of Pi16^+^ fibro‐progenitors was connected with the downregulation of VEGF‐C/D expression (**Figure** **5**A). To verify, we used TGF‐*β* to transdifferentiate Pi16^+^ fibro‐progenitors into POSTN^+^ myofibroblasts (Figure 5B,C). We noticed that the myo‐activated Pi16^+^ fibroblasts largely lost their VEGF‐C/D expression (Figure 5C). Likewise, when harvesting the AVFs from *Pi16*‐Cre^ER^;R26‐tdT mice, while the tdT^+^Pi16^+^ fibroblasts still possessed high levels of VEGF‐C expression, the tdT^+^POSTN^+^ fibroblasts lost such property (Figure 5D). For in vivo validation, we used *Pi16*‐Cre^ER^;R26‐tdT‐DTA mice to decrease Pi16^+^ fibro‐progenitor proportions in AVFs (Figure 5E and Figure S5H, Supporting Information). As the result, the lymphangiogenesis in AVFs was inhibited (Figure 5F), and the VEGF‐C/D factors also decreased (Figure 5G). For further determining which lymphangiogenic factor was more crucial in Pi16^+^ fibro‐progenitors, we constructed two conditional knockout mice, *Pi16*‐Cre^ER^;*Vegfc* ^fl/fl^ and *Pi16*‐Cre^ER^;*Vegfd* ^fl/fl^ mice (Figure 5H,I). After Tam treatment, although overall AVF fibrosis was insignificantly affected (Figure 5J), the wholemount staining manifested that *Vegfc* deficiency in Pi16^+^ fibro‐progenitors had a bigger influence on the AVF lymphatic network (Figure 5K). Above data indicate that, in AVFs, the VEGF‐C related lymphangiogenic function was another specific characteristic for Pi16^+^ fibro‐progenitors, which could be lost when experiencing myo‐activation.

### 2.5 5/6 Nephrectomy (Nx) Increased sVEGFR3 to Hinder VEGF‐C–Relied Lymphangiogenesis in Mouse AVFs

**Figure 6:**
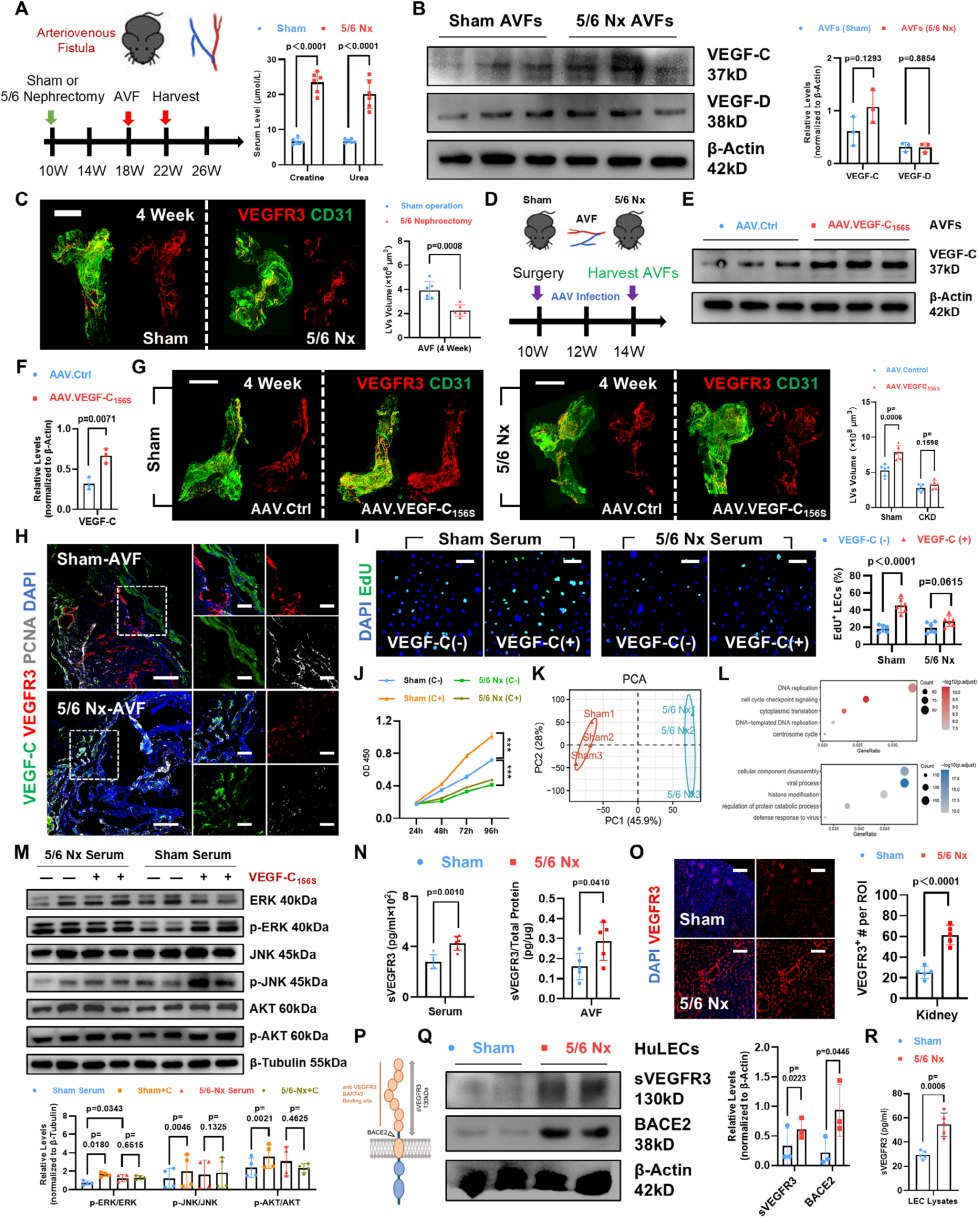
The defective lymphatic network with abnormal VEGF‐C responsiveness in mouse AVFs with 5/6 nephrectomy. A) Schematic diagram illustrating the mouse AVFs with sham operation or 5/6 nephrectomy (Nx); Evaluation of serum creatine or urea shown on the right (*n* = 6, unpaired two‐tailed Student's *t*‐test). B) Western Blot analyses of VEGF‐C and VEGF‐D in AVFs from sham or 5/6‐Nx mice; Quantifications shown on the right (*n* = 3, unpaired two‐tailed Student's *t*‐test). C) Representative whole‐mount staining for VEGFR3 (red) and CD31 (green) of AVFs from sham or 5/6‐Nx mice, with quantifications of lymphatic vessel volume (*n* = 6, unpaired two‐tailed Student's *t*‐test). D–F) Schematic diagram (D) and Western Blot analyses (E) of VEGF‐C in AVFs, with AAV.Ctrl or AAV.VEGF‐C_156S_ infection (*n* = 3, unpaired two‐tailed Student's *t*‐test); Quantifications shown in (F). G) Representative whole‐mount staining for VEGFR3 (red) and CD31 (green) of sham or 5/6‐Nx AVFs, with AAV.Ctrl or AAV.VEGF‐C_156S_ infection; Quantifications of lymphatic vessel volume (*n* = 6, unpaired two‐tailed Student's *t*‐test) shown on the right. H) Representative immunostaining for VEGFR3 (red), VEGF‐C (green), and PCNA (gray) in AVF sections from sham or 5/6‐Nx mice. I,J) Representative images of EdU assay (*n* = 6, unpaired two‐tailed Student's *t*‐test) (I) and CCK‐8 assay (*n* = 6, unpaired two‐tailed Student's *t*‐test) (J) in 5% sham or 5/6‐Nx serum treated HuLECs, with or without VEGF‐C_156S_ stimulation. K,L) Bulk RNA sequencing for VEGF‐C_156S_ treating HuLECs with 5% sham or 5/6‐Nx mouse serum (*n* = 3); Principal component analysis (PCA) plot shows the Intra‐group differences (K), and bubble plot shows the top enriched biological functions (L). M) Western Blot analyses of ERK, p‐ERK (Thr202/Tyr204), JNK, p‐JNK (T183/Y185), AKT, p‐AKT (ser473) of 5% sham or 5/6‐Nx serum treated HuLECs, with or without VEGF‐C_156S_ stimulation. Quantifications shown on the bottom (*n* = 3, unpaired two‐tailed Student's *t*‐test). N) Quantifications of serum or AVF sVEGFR3 in sham or 5/6‐Nx mice (*n* = 6, unpaired two‐tailed Student's *t*‐test). O) Representative immunostaining and quantifications (*n* = 5, unpaired two‐tailed Student's *t*‐test) for VEGFR3^+^ (red) LECs in remnant kidney from sham or 5/6‐Nx mice. P) Schematic diagram showing the VEGFR3 cleaved by BACE2 to release the soluble ectodomain, sVEGFR3 (130 kDa), the BAF743 antibody binding site. Q,R) Western Blot analyses with quantifications of sVEGFR3 and BACE2 (*n* = 3, unpaired two‐tailed Student's *t*‐test) (O), and ELISA analyses of sVEGFR3 (*n* = 5, unpaired two‐tailed Student's *t*‐test) (P) in 5% Sham or 5/6‐Nx serum treated HuLECs. Data are mean ± SD. Scale bars, 1000 µm in (C,G), 210 and 70 µm in magnification (H,I,O). *P* values of each comparison were specified in the graph. C+, VEGF‐C_156S_+.

Although above data has confirmed the critical role and cellular sources of VEGF‐C in mouse AVFs, the animal models were performed under wild‐type (WT) background. Unlike WT mice, although C57BL/6 mice are known to be resistant to the induction of experimental CKD by 5/6 Nx,^[^ ^38^ ^]^ previous studies have confirmed that 5/6 Nx could aggravate local inflammation and neointima formation in C57BL/6 mouse AVFs.^[^ ^9^, ^39^, ^40^ ^]^ Similarly, we created C57BL/6 mice with sham operation or 5/6 Nx, and performed cervical AVFs 8 weeks later (**Figure** **6**A). Compared with sham‐surgery mice, although serum creatinine and urea drastically increased in mice with 5/6 Nx (Figure 6A), we did not find significant differences between their VEGF‐C/D expression in AVF tissues (Figure 6B). However, while the lymphangiogenesis was still evident in mouse AVFs with 5/6 Nx, their lymphatic density was significantly lower than sham AVFs (Figure 6C). In order to exclude the false‐negative results by only Western Blot analysis, we used AAV to overexpress a selective VEGFR3 stimulant,^[^ ^41^ ^]^ VEGF‐C_156S_, in mouse AVFs (Figure 6D–F). As the result, the lymphangiogenesis in AVFs from sham mice was perfectly promoted, whereas the lymphatic regeneration in 5/6‐Nx AVFs did not receive similar effects (Figure 6G). Adjacent to the VEGF‐C expressing area, AVF LECs in 5/6‐Nx mice appeared more discontinuous structures and lower proliferating cell nuclear antigen (PCNA) expression (Figure 6H). More importantly, the serum extracted from 5/6‐Nx mice largely inhibited the pro‐proliferation effect by VEGF‐C_156S_ in HuLECs. By contrast, serum from sham mice had an insignificant effect (Figure 6I,J). We compared the VEGF‐C_156S_‐treating LECs with either sham or 5/6‐Nx serum by bulk RNA sequence. After principal component analysis (PCA) (Figure 6K), we confirmed several functional enrichments essential for cellular proliferation, such as DNA replication or cell cycle signaling, were inhibited in HuLECs treated with 5/6‐Nx mouse serum (Figure 6L). These results indicate that the less responsiveness to VEGF‐C is probably a crucial factor leading to the impaired lymphatic network in 5/6‐Nx mouse AVFs.

To provide reasonable explanation, we first checked the major downstream pathways activated by VEGF‐C/VEGFR3 signaling in HuLECs. We observed that VEGF‐C_156S_ expectedly activated ERK1/2, AKT, and JNK phosphorylation,^[^ ^29^, ^42^ ^]^ whereas a broad‐spectrum inhibition happened when 5/6‐Nx serum was added into the medium (Figure 6M). However, none specific downstream pathway was found to be uniquely responsible for the VEGF‐C unresponsiveness induced by 5/6‐Nx serum. We speculate that there may be some obstacles that prevent VEGF‐C from binding to the VEGFR3 under 5/6 Nx condition. Following this clue, we detected both systemic (serum) and local (AVF) elevations of sVEGFR3 in 5/6‐Nx mice (Figure 6N). In view of that sVEGFR3 is produced by the cleavage of VEGFR3 at juxtamembrane domain,^[^ ^24^ ^]^ we compared the VEGFR3^+^ LEC density in multiple organs between sham and 5/6‐Nx mice. In accordance with previous studies,^[^ ^43^ ^]^ we found that the lymphatic density increased in remnant kidney (Figure 6O). However, considering that the AVF tissue was harvested without circulation proteins, and VEGFR3^+^ lymphatic network was less‐formed in 5/6‐Nx AVFs, we reckoned that the local sVEGFR3 increment in 5/6‐Nx AVFs was more likely to be induced by enhanced VEGFR3 cleavage other than increased VEGFR3 amount. To validate, despite the indetectable sVEGFR3 in the medium, we used a specific antibody, BAF743, to detect the 130 kDa‐size extracellular domain of VEGFR3 in HuLEC lysates, which represented the sVEGFR3 fragment (Figure 6P). After normalization, we found increased sVEGFR3 was generated in HuLECs treated with 5/6‐Nx serum compared with sham serum, re‐confirmed by ELISA assay (Figure 6Q,R). These results show that systemic sVEGFR3 in 5/6‐Nx mice may be related to the increased LEC density in remnant kidney, but the enhanced VEGFR3 cleavage must contribute to the increased sVEGFR3 in AVF tissue.

### 2.6 Inhibiting BACE2 Mediated VEGFR3 Cleavage Normalizes the Lymphatic Network in AVFs with Hyperphosphatemia

**Figure 7:**
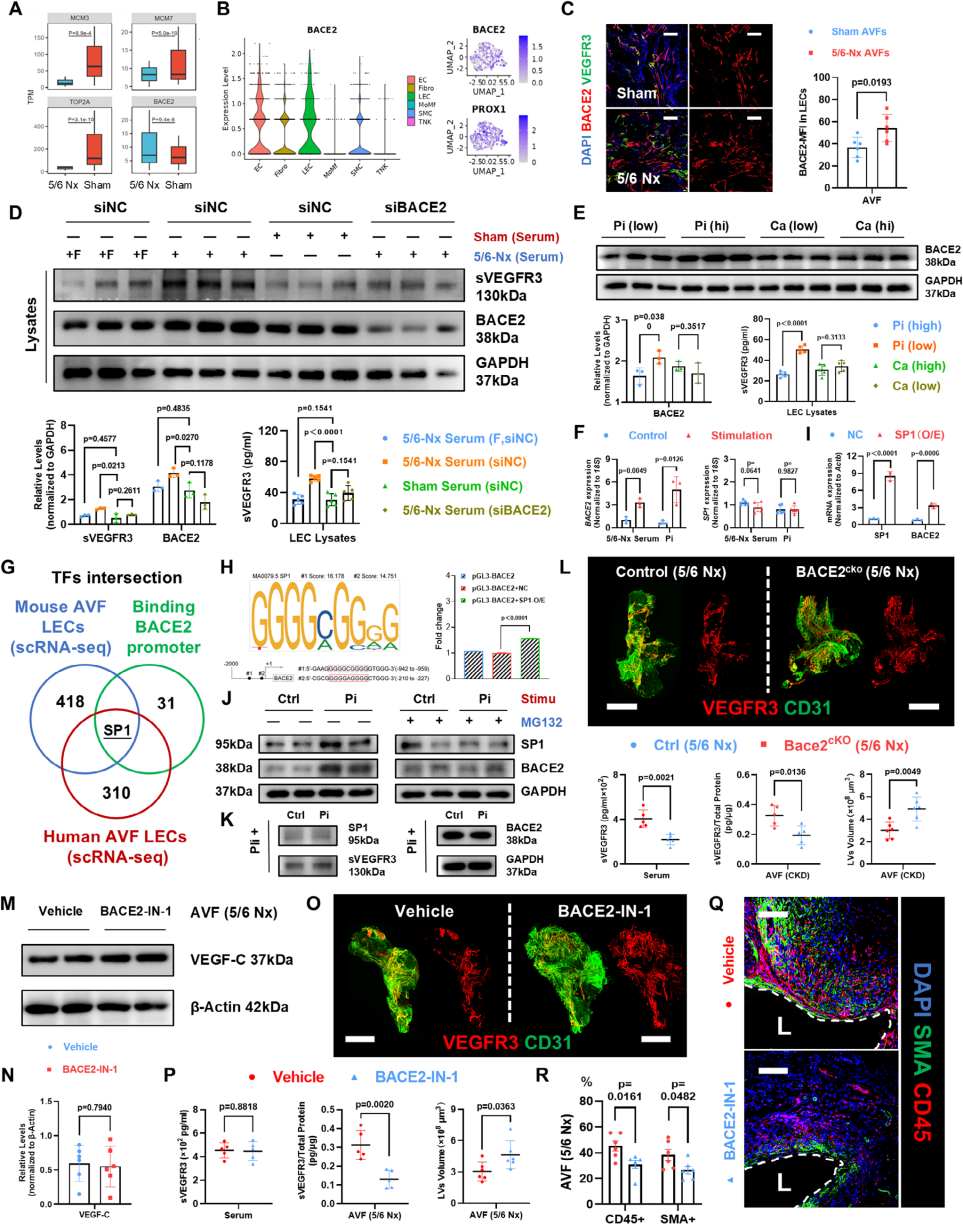
Inhibiting BACE2 reduced sVEGFR3 to normalize the lymphatic network in mouse AVFs with hyperphosphatemia. A) Box plot showing the comparisons of proliferation genes (*MCM3*, *MCM7*, *TOP1*), and *BACE2* between VEGF‐C_156S_ treating HuLECs added with sham or 5/6‐Nx serum from Bulk‐seq (*n* = 3, unpaired two‐tailed Student's *t*‐test). B) Violin plot showing *BACE2* expression in all cell types from human AVFs, and feature plot showing *BACE2* and *PROX1* expression in AVF LECs (scRNA‐seq). C) Representative immunostaining for BACE2 (red) and VEGFR3 (green) in AVF sections from sham or 5/6‐Nx mice, with quantifications of mean fluorescence intensity (MFI) of BACE2 in LECs (*n* = 6, unpaired two‐tailed Student's *t*‐test). D) Western Blot analyses with quantifications of sVEGFR3 and BACE2 expression, and ELISA analyses of sVEGFR3 in HuLEC transfected with small interfering RNA negative control (siNC) or siBACE2, treated with 5% sham serum or 5/6‐Nx serum or filtered 5/6‐Nx serum (*n* = 3–5, unpaired two‐tailed Student's *t*‐test). E) Western Blot and ELISA analyses of HuLECs treated with low or high phosphate (2/4 µmol L^−1^ Na_2_HPO_4_+NaH_2_PO_4_ (1:1)) or calcium (2/3 µmol L^−1^ CaCl_2_) stimulation (*n* = 3–5, unpaired two‐tailed Student's *t*‐test), according to the electrolyte differences between sham and 5/6‐Nx mouse serum (Figure S6B, Supporting Information). F) Quantitative PCR analyses of *BACE2* and *SP1* in HuLECs, treated with control (sham serum or low Pi) or stimulations (5/6‐Nx serum or high Pi) (*n* = 3 or 6, unpaired two‐tailed Student's *t*‐test). G) Veen plot showing the intersection of transcription factors (TFs) expressed by LECs in human or mouse AVFs, and TFs predicted to bind to *BACE2* promoters in JASPAR dataset. H) SP1‐BACE2 binding consensus sequence, with analysis of BACE2 promoter activity in the luciferase reporter assay of HuLECs, cotransfected with the BACE2 promoter‐pGL–basic plasmid and NC or SP1 plasmid (SP1‐O/E) (*n* = 3, one‐way ANOVA followed with Tukey's post hoc analysis). I) Quantitative PCR analyses of *BACE2* and *SP1* in HuLECs, transfected with negative control (NC) or SP1 plasmid (SP1‐O/E) (*n* = 3, unpaired two‐tailed Student's *t*‐test). J) Western Blot analyses of SP1 and BACE2 changes in HuLECs in response to Pi stimulation, with or without MG132 treatment; Quantifications shown in the Figure S6D (Supporting Information). K) Western Blot analyses of BACE2 changes in HuLECs in response to Pi stimulation, with or without SP1 inhibitor (Plicamycin) treatment; Quantifications shown in the Figure S6E (Supporting Information). L) Representative whole‐mount staining for VEGFR3 (red) and CD31 (green) of 5/6‐Nx AVFs from Tam‐treated control (*Prox1*‐Cre^ER^) or *Prox1*‐Cre^ER^;*Bace2* ^fl/fl^ (*Bace2* ^cKO^) mice, with corresponding quantifications of lymphatic vessel volume, serum or AVF sVEGFR3 (*n* = 5–6, unpaired two‐tailed Student's *t*‐test). M,N) Western Blot analyses and quantifications of VEGF‐C in AVFs from 5/6‐Nx mice, treated with vehicle control or BACE2‐IN‐1 (*n* = 6, unpaired two‐tailed Student's *t*‐test). O,P) Representative whole‐mount staining for VEGFR3 (red) and CD31 (green) of 5/6‐Nx AVFs from WT mice treated with vehicle or BACE2‐IN‐2 (O), with corresponding quantifications of lymphatic vessel volume, serum or AVF sVEGFR3 (*n* = 5–6, unpaired two‐tailed Student's *t*‐test) (P). R,Q) Quantifications of SMA^+^ or CD45^+^ fractions (*n* = 6, unpaired two‐tailed Student's *t*‐test) (R), with representative immunostaining for CD45 (red) and SMA (green) in 5/6‐Nx AVFs from WT mice treated with vehicle or BACE2‐IN‐2 (Q). Data are mean ± SD. Scale bars, 1000 µm in (L,O), and 210 µm in (C,Q). *P* values of each comparison were specified in the graph. +F, filtered 5/6‐Nx serum; Pi, phosphate; Stimu, stimulation; and Pli, Plicamycin.

Having confirmed the increased sVEGFR3 under 5/6‐Nx condition, we searched for the inducement. After checking the differentiated genes in bulk RNA sequencing (Figure 6K,L), except for several proliferation‐related genes (*MCM3*/*MCM7*/*TOP1*), we found a reported VEGFR3 cleavage enzyme,^[^ ^24^ ^]^ *Bace2*, was more highly‐expressed in 5/6‐Nx serum treated HuLECs (**Figure** **7**A). Using scRNA‐seq data from human AVFs, we found that despite BACE2 was widely expressed in various cells, it was most highly and endogenously expressed by LECs in AVFs (Figure 7B). Crucially, when comparing AVF tissues from sham or 5/6‐Nx mice, although we did not find significant discrepancy between their overall BACE2 expression (Figure S6A, Supporting Information), the LECs in 5/6‐Nx AVFs tended to possess higher BACE2 signal strengths (Figure 7C). For in vitro validation, when 5/6‐Nx serum was added into culturing medium, the BACE2 expression was largely upregulated in HuLECs (Figure 6R). When using small interfering RNA to specifically knockdown BACE2 in HuLECs, 5/6‐Nx serum lost its auxo‐action in the sVEGFR3 generation (Figure 7D).

How 5/6‐Nx serum affects the BACE2 expression in LECs? In consideration of the complexity in mouse serum, we first used ultrafiltration centrifuge tube to separate the proteins (over than 10 kDa) from mixed components in serum. Unexpectedly, the proteins extracted from 5/6‐Nx serum did not show similar upregulation effect on BACE2 and sVEGFR3 in HuLECs (Figure 7D). By contrast, among the most representative electrolyte disturbances in 5/6‐Nx mice (Figure S6B, Supporting Information), high phosphate (Pi) but not calcium (Ca) upregulated BACE2 and sVEGFR3 expression in HuLECs (Figure 7E), which resembled the 5/6‐Nx serum effect to a greater extent. When we balanced the Pi concentrations between sham and 5/6‐Nx serum, the upregulated BACE2 induced by 5/6‐Nx serum disappeared (Figure S6C, Supporting Information). These results indicate that the high Pi is the determinant in 5/6‐Nx serum regulated BACE2 upregulation.

Mechanistically, considering that the upregulated *Bace2* was embodied on the transcriptional level (Figure 7F), we selected transcriptional factor (TF) intersection between the TFs able to bind BACE2 promoter using TRRUST database, and TFs simultaneously expressed in human and mouse AVF LECs using scRNA‐seq. Resultantly, an activating TF, *SP1*, was revealed as a candidate (Figure 7G). Using dual‐luciferase reporter system, we confirmed that the SP1 activated the promoter area of BACE2 (Figure 7H), and overexpression of *SP1* increased *BACE2* transcription (Figure 7I). Using Western Blot analyses, we found that the SP1 content increased after Pi stimulation (Figure 7J and Figure S6D, Supporting Information). We combined the Pi stimulation with a SP1 inhibitor, Plicamycin, and found that the Pi‐induced BACE2 and sVEGFR3 upregulation was suppressed (Figure 7K and Figure S6E, Supporting Information). However, we found the transcriptional level of *SP1* was not upregulated in response to 5/6‐Nx serum or high Pi (Figure 7F), suggesting that the posttranscriptional modification may be the mechanism. Previously, the phosphorylation of TFs has been confirmed as the major intracellular signals in response to the elevated inorganic Pi.^[^ ^44^ ^]^ Similarly, we detected the phosphorylated SP1 (p‐SP1) was activated in HuLECs when sensing extra Pi, especially at Thr278 (Figure S6F, Supporting Information). Having proved that p‐ERK signals increased in 5/6‐Nx serum treating LECs (Figure 6M), which dominantly regulate p‐SP1 in multiple cell lines,^[^ ^45^, ^46^ ^]^ we used AG126, the p‐ERK inhibitor, and found that the p‐SP1 modification induced by Pi stimulation was inhibited (Figure S6F, Supporting Information). The Thr278 phosphorylation of SP1 has been reported to stabilize SP1 through inhibiting the proteasome degradation,^[^ ^45^ ^]^ we used MG132, a proteasome inhibitor, and found that the Pi‐caused SP1 accumulation and BACE2 upregulation were counteracted in HuLECs (Figure 7J and Figure S6D, Supporting Information). Above results indicate that Pi tends to activate the phosphorylation of ERK/SP1 in sequence, which stabilize the SP1 to enhance BACE2 expression in LECs.

Took above message into account, we constructed a LEC specific BACE2 knockout mice, *Prox1*‐Cre^ER^;*Bace2* ^fl/fl^ (*Bace2* ^cKO^) (Figure S6G, Supporting Information). After Tam treatment, compared with control mice (*Prox1*‐Cre^ER^), BACE2 was completely silenced in AVF LECs without compromised kidney function (Figure S6H,I, Supporting Information). Concomitantly, the systemic and local sVEGFR3 were largely decreased, and the defective lymphangiogenesis was recovered in 5/6‐Nx AVFs from *Bace2* ^cKO^ mice (Figure 7L). In addition, the CD45‐representing inflammatory infiltration and intimal hyperplasia were alleviated (Figure S6J, Supporting Information). Gaining insights from above phenotypes, we applied BACE2 inhibitor, BACE2‐IN1, in the anastomotic area to decrease the BACE2 activity without changing VEGF‐C expression in mouse AVFs with 5/6 Nx (Figure 7M,N). Essentially, although systemic sVEGFR3 was not affected, the lymphatic network was recovered when local sVEGFR3 were decreased (Figure 7O,P). Concomitantly, the CD45^+^ fractions and neointima were reduced in 5/6‐Nx AVFs (Figure 7Q,R).

## 3 Discussion

Due to the lower infection rates and less complications, successfully mature AVF is undoubtedly a preferred method for vascular access in hemodialysis ESRD patients. Since clinical^[^ ^4^, ^5^, ^6^ ^]^ and animal studies^[^ ^7^, ^8^ ^]^ have both indicated the importance of inflammatory reaction in AVF failure, seeking novel immunoregulation mechanisms and targets is always an essential topic in AVF studies. Distinct from traditional perspectives, such as endothelial protection^[^ ^47^ ^]^ or hemodynamics adjustment,^[^ ^48^ ^]^ we pointed out that the adventitial lymphatic network was another overlooked structure. Lymphatic vessels serve as the highly‐permeable outflow tunnel for immune cells in AVFs, and a defective lymphatic network would aggravate the local inflammation in AVFs. It is worth noting that AVF remodeling still occurs in the absence of lymphatic growth, suggesting that the role of lymphatics may be modulatory rather than indispensable, and alternative mechanisms for inflammatory efflux in AVFs are not ruled out. Particularly, in mouse AVFs with hyperphosphatemia, we found that BACE2 inhibition could rectify the sVEGFR3‐related VEGF‐C unresponsiveness in LECs, and normalized the lymphatic network to alleviate local inflammation and neointima formation. Since neointima is present preoperatively in human veins and does not predict AVF failure,^[^ ^49^, ^50^, ^51^ ^]^ the neointima phenotype here was more like another indicator to evaluate the inflammation severity in mouse AVF remodeling. Next, we chose several essential points to discuss in detail.

When using spatial transcriptomics and scRNA‐seq techniques to portray the cellular landscapes of AVFs across species, evident fibrosis was revealed as one of the conservative features after fistulation, among which a group of Pi16^+^ fibroblasts caught our attention. Previously, Pi16^+^ fibroblasts were regarded as progenitor‐like fibroblasts that developed into other specialized fibroblasts,^[^ ^36^, ^52^ ^]^ and also enriched with some unique properties, including potential anti‐tumor functions^[^ ^53^ ^]^ or T cell regulation.^[^ ^54^ ^]^ We found that a fluctuant lymphangiogenic property was another special characteristic of this cluster. Specifically, while Pi16^+^ fibro‐progenitors could be activated by inflammatory cytokines, such as TNF‐*α*, and largely upregulate their VEGF‐C/D expression to enhance lymphangiogenic ability, they could also differentiate into myofibroblasts and lose the advantageous lymphangiogenic property. This dynamic transformation embodies that the lymphangiogenesis in AVFs tends to be a self‐limiting process, as the increased cytokines have bidirectional effects on the lymphangiogenic fibroblasts and therefore avoid limitless lymphangiogenesis.

Contrary with the LECs efficiently receiving VEGF‐C stimulation under normal condition, we demonstrated that the lymphatic network in AVFs with hyperphosphatemia was insensitive to VEGF‐C signals. Previously, CKD has been reported to complicate mouse AVF remodeling by multiple ways, including aggravating endothelial dysfunction,^[^ ^9^ ^]^ promoting EndMT^[^ ^20^ ^]^ or stem cells differentiation,^[^ ^40^ ^]^ but not substantiated by current human data. In this study, we discovered that the hyperphosphatemia, one of the common metabolic disorders in pre‐dialysis CKD patients, impeded the construction of mouse AVF lymphatic network by producing additional sVEGFR3, the decoy receptor for VEGF‐C, and blunted VEGF‐C responsiveness in LECs. Due to this weakness, unlike other vascular diseases,^[^ ^16^, ^17^ ^]^ simply providing extra VEGF‐C sources could not effectively promote the lymphangiogenesis in AVFs with hyperphosphatemia. It is noteworthy that our AVFs were created under the established hyperphosphatemia status, therefore it is not controversy to detect increased lymphangiogenesis in kidney^[^ ^43^ ^]^ or intestine^[^ ^55^ ^]^ during the progression of CKD. Moreover, whether the released sVEGFR3 affect other vascular beds in AVFs still require further validation.

Having known that sVEGFR3 shows a fast and sensitive response to BACE2 activity,^[^ ^24^ ^]^ we uncovered a significant upregulation of BACE2 in LECs with hyperphosphatemia. Previously, phosphorus level has been recognized as a risk factor for AVF dysfunction,^[^ ^56^ ^]^ but the mechanisms were unknown. In this study, we found that Pi activates the ERK/p‐ERK pathway and promotes SP1 stability by Thr278 phosphorylation in LECs, which eventually induces the intracellular accumulation of SP1. The increased SP1 binds to the promoter area and upregulate an essential VEGFR3 cleavage enzyme,^[^ ^24^ ^]^ BACE2, which cleaves VEGFR3 to release an immature and soluble ectodomain of VEGFR3, sVEGFR3, and competitively inactivate the VEGF‐C/VEGFR3 pathway in AVFs. By creating LEC‐specific BACE2 knockout mice or using BACE2 inhibitors, we observed that the lymphatic network was largely recovered in AVFs with hyperphosphatemia. Previously, BACE2 has more been reported as the sheddase for pigment cell‐specific melanocyte protein^[^ ^57^ ^]^ or Tmem27 in *β* cell,^[^ ^58^ ^]^ therefore discussed as a potential drug target for melanoma metastasis or diabetes treatment. In our study, we put forward an innovative application of BACE2 inhibitors, which aimed at reducing Pi‐induced VEGFR3 cleavage to reduce AVF local inflammation. Considering that overt hyperphosphatemia always inevitably occurs during late CKD progression,^[^ ^59^ ^]^ inhibiting BACE2 may become one of the promising therapeutic strategies. Furthermore, since the earliest lymphangiogenesis always appears at the anastomotic site of AVFs, we proved that applying BACE2 anastomotically was an appropriate adjustment to enhance the feasibility without affecting lymphatic circulation in other areas, although the sustaining effect needs more long‐term evaluation.

Our study has few limitations. First, due to significant histological differences (human veins are multilayered with valves, whereas mouse veins consist of a single layer of SMCs and ECs and lack valves), the mouse AVF model does not recapitulate the full spectrum of human venous adaptation to arterial flow. Second, ESRD and uremia alone do not predict AVF failure, and it is common for both functioning and failing segments to coexist within the same fistula from a single patient. Therefore, the role of CKD in AVF failure need to be further supported by robust evidence. Third, our evaluation of lymphatic architecture in AVFs was basically based on the volume, but not more detailed histological or molecular data.

## 4 Experimental Section

### Human Venous Sample Collection

Cephalic veins were collected from patients during their first fistulization. Venous segments of AVFs were collected from patients who underwent renal transplant and volunteered to remove AVF structures. The selection of patient samples was primarily determined by clinical availability. All patients had signed informed consent form before sample collection, and the study protocol was approved by the Clinical Research Ethics Committee of the First Affiliated Hospital, School of Medicine, Zhejiang University (IIT20210018B‐R2). Detailed information of all patients is listed in Table S1 (Supporting Information).

### Mouse Generation and Breeding

*Lyve1*‐Cre^ER^ knock‐in mouse model was developed by Shanghai Model Organisms Center, Inc. as described previously.^[^ ^19^ ^]^ Wildtype (C57BL/6) mouse lines were purchased from Shanghai SLAC Laboratory Animal Co., Ltd. *Pi16*‐Cre^ER^, *Prox1*‐Cre^ER^, *Vegfc* ^em1Cflox^, *Vegfd* ^em1Cflox^, and *Bace2* ^em1Cflox^ mouse lines (C57BL/6) were purchased from GemPharmatech Co., Ltd. Other mouse lines including, R26‐tdTomato (JAX: 007909) and R26‐DTA (JAX: 006331) mice lines were purchased from Shanghai Model Organisms Center, Inc. Genomic DNA from mice tail tissue was prepared by proteinase K lysed, isopropanol precipitated and 70% ethanol washed and then prepared for genotyping.

All murine experiments were approved by The Tab of Animal Experimental Ethical Inspection of the First Affiliated Hospital, Zhejiang University school of Medicine (No. 2020 (105)). The sample size for animal experiments was predetermined with a significance threshold (*α*) value of 0.05 and a power (1−*β*) value of 0.8 on the basis of these preliminary experiments. Male and female mice were both used and were randomly allocated to different experimental groups. The numbers of mice were indicated in the figure legends for each experiment. All mice generated or purchased were housed in Laboratory Animal Center of Zhejiang University (Hangzhou, China) for at least 1 week before use. The animals were excluded if they accidently died pre‐harvesting, preventing the collection of histological data.

### The Cervical Mouse AVF Models

The basic protocols for mouse cervical AVF models were performed as described previously.^[^ ^25^ ^]^ Generally, mouse AVFs were constructed by an end (vein)‐to‐side (artery) anastomosis between left carotid arteries and external jugular veins (JVs) using interrupted stitches of Ethilon 11‐0 sutures. The cut portions of the external JVs were ligated using Ethilon 8‐0 sutures. After AVF procedure, prior to closing the surgical site, blood flow and pulsation of the AVFs was visually assessed to confirm the success of fistula formation.

### The 5/6 Nephrectomy Model of Mouse

For C57BL/6 mice with 5/6 nephrectomy, a two‐step procedure was performed as described previously.^[^ ^26^ ^]^ Briefly, 1/3 of each pole of the left kidney was ligated with a silk suture and excised with scissors. Special care was taken to avoid damage to the adrenals. The entire right kidney was removed 1 week later to complete the 5/6 nephrectomy. Sham‐operated control mice were subjected to a similar procedure without removal of kidney tissue.

### RNA Extraction, Reverse Transcription, and Quantitative Polymerase Chain Reaction

Total RNA was extracted from venous tissues or cultured cells using TRIzol reagent. A RevertAid RT Reverse Transcription Kit (Thermo Fisher Scientific, 15596018) was used for reverse transcription of mRNA to cDNA and further amplification. Quality control of samples was performed using a Nanodrop ND‐1000 spectrophotometer. Quantitative polymerase chain reaction (qPCR) of mRNA products was performed using a TB green system, and each measurement was carried out in duplicate using a ViiA 7 real‐time PCR system. Primer sequences used for qPCR in this study are listed in Table S2 (Supporting Information).

### Statistical Analysis

Blinding was used for each step in the experimental process. Numbers (*n*) refer to the number of independent experiments or mice which were indicated in relevant figure legends. Data are presented as mean ± SD. For animal data, data was first analyzed for normality test by D'Agostino and Pearson (*n* = 6) or Shapiro–Wilk (*n* = 3–6) test. Between two groups, data was tested by unpaired or paired two‐tailed *t*‐test, with Welch's correction if uneven variances existed. For over two groups, abnormal distribution data was firs*t*‐tested by non‐parametric Kruskal–Wallis test with Dunn's post‐hoc test, and other normal distribution data were tested by ordinary one‐way ANOVA with Tukey test (equal SDs) or by Brown–Forsythe and Welch ANOVA tests with Dunnett's T3 test (significant different SDs). For cell culture data, data was firs*t*‐tested by Shapiro–Wilk test and then assessed by unpaired two‐tailed *t*‐test. *P* < 0.05 was considered to be statistically significant. Statistical methods used to assess significant differences with sufficient details are shown in figure legends.

Detailed methods are provided in the Supporting Information.

## Conflict of Interest

The authors declare no conflict of interest.

## Author Contributions

K.C. and Z.G. contributed equally. Conceptualization: K.C. and Q.X.; Methodology: K.C., Z.G., H.J., M.A., R.M., M.Y., and Y.H.; Investigation: K.C., L.J., X.W., H.Y., Y.L., and T.C.; Formal Analysis: K.C. and Z.G.; Resources: K.C., Q.X., H.Z., and W.L.; Writing—Original Draft: K.C.; Writing—Review and Editing: Q.X., H.Z., and W.L.; Supervision: Q.X.

## Supporting information



Supporting Information

## Data Availability

The data that support the findings of this study are available on request from the corresponding author. The data are not publicly available due to privacy or ethical restrictions.
