# Supplementary material for: BACE2‐Induced Aberrant Lymphatic Network Aggravates the Local Inflammation in Arteriovenous Fistulas With Hyperphosphatemia
Source: Adv Sci (Weinh). 2025 Aug 30;12(43):e09632. doi: 10.1002/advs.202509632 (PMC12631814; doi:10.1002/advs.202509632)
Supplement: Supplementary file 1 — Supporting Information [file ADVS-12-e09632-s001.pdf]

METHODS

Lymphangiogenesis Intervention in Mouse AVFs

For mice treated with adeno-associated virus (AAV) infection, $VEGFC_{156S}$ or sVEGFR3 vectors were produced as described[1,2]. Serotype 5 was selected. AAV with empty vectors was treated as control. Specifically, AAV was firstly prepared by mixing AAV ($5x10^{12}$ vg/ml) with 25% Pluronic F-127 Gel (PF-127, Sigma) for 30 minutes at 4 °C. After the cervical AVFs were constructed, about $1x10^{12}$ vg/ml AAV was instantly delivered to the adventitial side of AVFs. In order to prevent the autofluorescence to interfere with the flow cytometry or immunostaining results, the used AAV were created without autofluorescence. To investigate the effect of BACE2 inhibition on AVF lymphangiogenesis, 40 μg BACE2-IN-1 (MCE, HY-136742) mixed with PF-127 gel was delivered onto the AVFs from 5/6-Nx mice immediately after surgery.

Tamoxifen-induced Genetic Labeling or Conditional Knockout in mice

To induce the fluorescent protein in $PROX1^+$, $LYVE1^+$ or $PI16^+$ cells from *Prox1*-Cre^{ER};R26-tdT, *Lyve1*-Cre^{ER};R26-tdT or *Pi16*-Cre^{ER};R26-tdT mice, or conditional knockout *Vegfc* or *Vegfd* in $Pi16^+$ cells from *Pi16*-Cre^{ER};*Vegfc*/*Vegfd*$^{fl/fl}$ mice, or conditional knockout *Bace2* in LECs from *Prox1*-Cre^{ER};*Bace2*$^{fl/fl}$ mice, tamoxifen (Sigma, T5648, 0.15 mg/g body weight) dissolved in corn oil (Sigma, C8267, 20 mg/ml) was administered by gavage every 3 days with a total of 4 pulses.

Isolation of Single Cells from Human and Mouse Vessels

Human and mouse veins or venous segments of AVFs were harvested, washed with PBS, and placed in a petri dish containing DMEM (Sigma, D6429) with 10% fetal bovine serum (FIBROBLASTS, HyClone, SH30396.03) on ice. Before digestion, tissue was cut into fine pieces in PBS, and floating adipose tissue was abandoned. The sediment was digested at 37°C water bath with reciprocal shaking in a mixed digesting solution contacting papain, collagenase I and

dispase II. All digested cells from one sample were pooled together at the end of the digestion process. After sufficient digestion, cells were filtered through a 40-μm cell strainer, and collected through centrifugation. Subsequently, cell pellet was suspended in relevant solutions for further experiments.

Flow Cytometric Analyses.

For digested mouse tissue, collected cells were incubated with red blood cell lysis buffer (Solarbio, R1010) for 5 minutes to lyse erythrocytes and passed through 40-μm cell strainer to obtain single-cell suspensions, which could be directly analyzed for autofluorescence. For the staining of certain cell surface markers, cells were stained with conjugated-antibodies (1 μg per 10^6 cells) for 30 min, LIVE/DEAD™ Near-IR Stain Kit (Invitrogen, L34975) for 10 mins, and then washed and re-suspended in PBS containing 0.1% FIBROBLASTS for flow cytometric analyses. The primary antibodies included CD45-FITC (Invitrogen, 11-0451-85), VEGFR-3-PE (R&D systems, FAB743P), Streptavidin-APC (Invitrogen, 17-4317-82), LYVE1-PE-Cy7 (Invitrogen, 25-0443-82), BACE2-Biotin (Invitrogen, BACE2-BIOTIN). All prepared samples were analyzed by BD LSR Fortessa II flow cytometer and FlowJo software. The exact cell numbers were calculated by total cell counting multiply with corresponding proportions shown in the flow cytometric analyses.

Spatial Transcriptomics RNA Sequencing

Human cephalic veins (n=2) and AVF samples (n=2) were embedded using OCT and cut into 10 um thick frozen sections and stored at -80°C. The 10x Genomics Visium spatial transcriptome platform based on poly-A captured mRNA was used for spatial transcriptome sequencing. Sections were imaged and processed for spatially resolved gene expression using 10x Genomics Visium Spatial Gene Expression Slide & Reagent Kit (PN-1000184) and Visium Spatial Gene Expression Slide Kit (PN-1000185). Libraries were sequenced on the Illumina NovaSeq platform and the resulting data were processed using

“SpaceRanger” (v.1.2.0), mapping to a reference human genome (GRch38-2020-A). Qualified DNA libraries were subjected for high-throughput sequencing using PE150 mode, with 499 spots in cephalic veins and 1825 spots in AVFs sequenced (mean reads of 259957 and 92577 per spot, as well as 15652 and 17048 total genes detected per spot).

Single-cell RNA sequencing

For single cell RNA sequencing (scRNA-seq), human cephalic veins (n=2), human AVFs (n=2), mouse jugular veins (n=10) and mouse AVFs (n=10) were obtained. As for pooling strategy, cells from Veins and AVFs was blended respectively. LIVE/DEAD and Hoechst staining was performed to sort living cells from mouse or human with nucleus during fluorescence activated cell sorting (FACS). Sorted cells were then subjected to scRNA-seq. The 10x Genomics ChromiumTM Single Cell 3' Reagent Kit v3 chemistry was used and followed the standard protocol for reverse transcription, cDNA amplification, and DNA library construction. The library was sequenced on the Illumina Novaseq6000 PE150 platform with paired-end 150 bp sequencing strategy. To generate feature-barcode matrix from fastq file, raw data were processed using “CellRanger” (v6.0) with a reference of mouse genome (mm-10-2020-A). As a result, the total cells detected in the samples of human cephalic veins, human AVFs, mouse jugular veins and mouse AVFs were 15442, 16306, 10894, and 10024, and the total genes detected were 27845, 26794, 21594, and 22428.

Bulk RNA Sequencing

HuLECs was cultivated with 100ng/ml VEGF-C_{156S} (R&D systems, 752-VC) and 5% sham or 5/6-Nx mouse serums for 48 hours before total RNA was extracted. The RNA Nano 6000 Assay Kit of Bioanalyzer 2100 system was used to assess the integrity of total RNA. Ploy-T oligo-attached magnetic beads was used to enrich mRNA with poly-A tail. After the procedure of reverse

transcription, cDNA amplification and purification, the library was built for subsequent sequencing. Qualified libraries were sequenced on Illumina Novaseq platform and 150 bp paired-end reads were generated.

Data Analyses for RNA sequencing

For spatial and single cell RNA sequencing, subsequent data normalization and integration was performed in R (v4.2.3) with default parameter, using “SCTransform” function from Seurat package (v4.2.3), and “RunHarmony” function from harmony package (v0.1.1). Data reduction and clustering performed with the function of “RunTSNE”, “FindNeighbors” and “FindClusters” in sequence. Additionally, for single cell RNA sequencing, cell filtration was performed before SCT normalization. Genes expressed in at least 3 cells and cells with at least 200 detected genes were kept, followed by filtering cells having over 5% mitochondrial counts. DoubletFinder (version 2.0.3) was applied to remove suspected double cells.

To integrate single cell or spatial transcriptomic sequencing data, cell2location (v0.1) was run in Python (v3.10), which predicts the cell types abundant in each spot. Cell types identification of spots was determined by the maximum abundant of cell types predicted. Layers of spots were clustered manually based on histologic location of vessels in HE staining. To quantify the layer location of spots, “spatial score” was calculated with a custom formula (Figure S1E). LOESS (locally weighted regression) was using to fit the curve associated with “spatial score”. Differential expression genes (DEGs) was generated with “Seurat::FindMarkers” function with logfc threshold as 0.25. Gene enrichment analysis was performed using the function of “enrichGO”, “enrichKEGG” from clusterProfiler package (v4.6.2). All DEGs were calculated with logfc threshold as 0, which used in GSEA (v1.2) analysis. GSVA (v1.46.0) analysis was performed with gene sets of “Hallmarker” and “C5” from msigdbr (v7.5.1) database. “Seurat::AddModuleScore” function was used to score specific gene sets. Communications and receptor ligand pairs analysis among

spots or single cell clusters were performed using CellChat package (v1.6.1). Trajectory inference was performed using monocle2 package (v2.28.0), and CytoTRACE package (v0.3.3) was used to predict the origin of the differentiation trajectory with high potential of cell differentiation. RNA velocity analysis followed the pipeline of scvelo package (v0.2.5) in Python (v3.10). Pseudo-bulk data was generated using "AverageExpression" function grouping by cell types. 2000 high variable genes were selected for pearson correlation analysis among cell types. 'AddModuleScore' function in the Seurat package (v4.2.3) was used to calculate the lymphangiogenic score of the Fibro spots at different distance from the LEC spots. The input gene set used for calculation 'was from the 'GOBP_LYMPHANGIOGENESIS' entry in MSDB-C5 (Molecular Signatures Database C5 subset). All genes in the entry have been listed below (PDPN/CCBE1/CLEC14A/EPHA2/VASH1/FOXC1/FOXC2/FLT4/PTPN20/ SOX18/PPP3CB/PROX1/PTPN14/BMPR2/TIE1/VEGFA/VEGFC/ACVR2B/MI R9-1/ACVRL1). The Fibro spots was divided into 4 groups based on the distance from the LEC spots. In order to compare the difference of the lymphangiogenic score among these 4 groups, Kruskal-Wallis test was used to for non-parametric tests, followed by Nemenyi test to perform pairwise comparisons.

For bulk RNA sequencing, Hisat2 (v2.0.5) and samtools (v2.10.9) was used to convert fastq files to bam files. Rsubread package (v2.12.3) was used to read bam files and obtain RNA count expression matrix. DEGs were calculated by DESeq2 package (v1.38.3) with count matrix. In order to execute principal component analysis (PCA), count matrix was normalized as transcripts per million (TPM) matrix using edgeR (v3.40.2). PCA plot was drawn using the function of "fviz_pca_ind" from FactoMineR package (v2.8).

Immunofluorescence and Histology Staining

For tissue clearing and 3D-reconstruction, detailed protocol has been described[3]. Briefly, mouse tissue was harvested and immersed in CUBIC-L

solution at 37°C for 2 days. After PBS wash, the tissue was conjugated in the primary antibody solution and incubated at 4°C for 2 days, and incubated with the secondary antibodies at RT for 1 day. After that the tissue was immersed in CUBIC-R^{+} solution for transparency. Images were obtained using a Leica TCSSP8 DIVE confocal microscope and further reconstructed using the Imaris 9.0.1 (Bitplane) software.

For cross-sectional immunofluorescence staining, venous samples were first harvested and fixed in 4% paraformaldehyde, followed by dehydration in 30% sucrose solution until fully penetrated. Tissue was then embedded in optimum cutting temperature (Sakura), frozen at -80°C for storage, and cut into 5-μm sections. Cryosections were air-dried for 30 mins at RT, blocked and permeabilized in 5% donkey serum with 0.1% Triton X-100 for 1 h, stained with primary antibodies overnight at 4°C, and then incubated with Alexa Fluor-conjugated secondary antibodies (Invitrogen) for 1 h, followed by DAPI staining and mounting in the anti-fade mounting medium. Isotype control primary antibodies for each host species with secondary antibody used as negative controls to eliminate the background signal. Primary antibodies were used as listed: tdTomato (Rockland, 600-401-379, 1:500), tdTomato (SICGEN, AB8181-200, 1:50), CD31 (R&D, AF3628, 1:50), SMA-FITC (Sigma, F3777, 1:300), CD45 (R&D systems, AF114, 1:50), PCNA (Proteintech, 60097-1-Ig, 1:100), VEGFR-3 (R&D system, AF743, 1:50; Abcam, ab27278, 1:100), and BACE2 (Abcam, ab5670, 1:100), POSTN (R&D systems, AF2955, 1:100), VEGF-C (Abcam, Ab106512, 1:100), and Pi16 (R&D systems, AF4929, 1:100). Secondary antibodies were used as listed: donkey anti-rabbit IgG Alexa Fluor 555 (Invitrogen, A-31572, 1:500), donkey anti-rabbit IgG Alexa Fluor 647(Invitrogen, A-32795, 1:500), donkey anti-rat IgG Alexa Fluor 647 (Invitrogen, A-48272, 1:500), donkey anti-mouse IgG Alexa Fluor 488 (Invitrogen, A-21202, 1:500), donkey anti-goat IgG Alexa Fluor 555 (Invitrogen, A-32816, 1:500), donkey anti-goat IgG Alexa Fluor 647 (Invitrogen, A-21447, 1:500). Isotype control primary antibodies (Invitrogen, Cat No. 31933, 02-6102,

31903, 31245) for each host species were used as negative controls.

For haematoxylin and eosin (H&E) and Masson's trichrome staining, procedures were performed according to manufacturer's instruction. The Image J software (NIH, USA) was used for image analyses. The investigators were blinded to different groups when performing histology staining and analyzing the data.

Lymphatic Endothelial Cells Culturing, Validation and Stimulation

HuLECs were acquired and verified by Shanghai Zhong Qiao Xin Zhou Biotechnology Co.,Ltd (ZQY068). Cell lines were authenticated using Short Tandem Repeat (STR) analysis as described in 2012 in ANSIStandard (ASN-0002) by the ATCC Standards Development Organization (SDO) and the Match criteria for human cell line authenticationInt J Cancer (2013:132(11):2510-9). Results showed that human cross-contamination was not found in this cell line. Cell lines were authenticated using immunofluorescent staining (VEGFR-3, proteintech, 20712-1-AP), and reconfirmed by LYVE1, and PROX1 immunofluorescent staining.

Mouse fresh blood was required to stand at room temperature until coagulation, then centrifuged at 3000rpm for 15 min to retain the supernatant as serum. To remove impurities, serum was centrifuged at 12000rpm for 30min and the supernatant was retained again. To concentrate and filter circulating proteins, serum was filtered using an Amicon ultra (MERCK, UFC8010) ultrafiltration centrifuge tube. Chemical stimulations, including, Na_2HPO_4, NaH_2PO_4 (7558-79-4, 13472-35-0), and $Cacl_2$ (10043-52-4), were acquired from Sinopharm Chemical Reagent Co., Ltd.

Cell Transfection and Dual-luciferase Reporter System

Plasmid were transiently transfected into HuLECs using LipofectamineTM 2000 according to the manufacturer's instructions. To study SP1-BACE2 interaction, HuLECs were co-transfected with pGL-basic containing BACE2 promoter,

control reporter RL-TK plasmids, and empty plasmid (negative control, NC) or SP1 plasmid (pcDNA3.1-T2A-EGFP, Youbio Biological Technology Co., Ltd.). HuLECs supernatant was harvested to measure luciferase activity 48 hours post transfection by Dual-Luciferase Reporter Assay System kit (Beyotime) by SpectraMax M5 plate reader.

Western Blot and ELISA Analyses

Cultured cells or tissue samples were lysed in RIPA lysis buffer supplemented with phosphatase inhibitor tablets and protease inhibitor tablet. Quantification of protein concentration was performed by Enhanced BCA Protein Assay Kit. Equal amounts of total protein lysates for each sample were loaded and electrophoresed on SDS–polyacrylamide gel (Bio-Rad) and then transferred to a PVDF membrane, followed by a standard Western Blot procedure. Primary antibodies were used as listed: BACE2 (Abcam, ab5670, 1:1000), VEGFR-3 (R&D systems, BAF743, 1:1000), VEGF-C (Santa Cruz Biotechnology, sc-374628, 1:1000), VEGF-D (Abcam, ab155288, 1:1000), Pi16 (R&D systems, AF4929, 1:1000), POSTN (R&D systems, AF2955, 1:1000), ERK (CST, 4695, 1:1000), p-ERK (Thr202/Tyr204) (CST, 9101S, 1:1000), AKT (HUABIO, ET169-51, 1:1000), p-AKT (ser473) (HUABIO, ET1607-73, 1:1000), JNK (diagbio, db2618, 1:1000), p-JNK (T183/Y185) (CST, 4669T, 1:1000), SP1 (Santa Cruz, sc-420, 1:1000), p-SP1 (Thy278) (Invitrogen, PA5-106039, 1:1000), β-Tubulin (CST, 2128s, 1:1000), GAPDH (HUABIO, HA721131, 1:5000) and HRP-Conjugated β-Actin (Proteintech, HRP-66009, 1:2000). Membranes were detected by the enhanced chemiluminescence (ECL) method and the image was captured with Biospectrum Imaging System (UVP, Upland, CA).

Mouse serum or AVF homogenate concentrations of sVEGFR3 were measured via mouse sVEGFR3 ELISA Kit (Huabang Bio, ST-H13362). HuLECs concentrations of sVEGFR3 (100μL) were measured via human sVEGFR3 ELISA Kit (Shanghai Hengyuan Bio, HB3808-Hu). To evaluate the sVEGFR3 level in mouse AVF tissue, we first measured the concentrations of total

sVEGFR3 (ng) in AVF homogenate, and then used Enhanced BCA Protein Assay Kit to measure the concentrations of total protein (μg) in total AVF homogenate. The final sVEGFR3 levels were calculated by sVEGFR3/Total protein (ng/μg). All experiments were performed according to the standard protocols provided by manufacturers.

Fibroblast Isolation, Culturing, and Stimulation

Human or mouse primary fibroblasts were isolated from control veins or venous segments of AVFs using explant method. Specifically, the venous tissues were harvested to DMEM containing 20% fetal bovine serum (FBS), with the perivascular fat striping. Then, we cut the vessel wall longitudinally into 1mm^3 pieces in sterile 20% DMEM, and spread on the bottom of the T25 culture flasks evenly. Then, the medium was aspirated carefully. The T25 flasks were incubate upright at 37°C for 3 hours to promote the fibroblast migration before 5 ml medium addition. The tissue pieces were cultivated for 5 days without movement to obtain overgrown fibroblasts. For passage cultivation, fibroblasts were digested with 0.25% trypsin and cultured with 10% DMEM in subsequence. fibroblasts at passage 2-3 were identified by COLLAGEN I and VIMENTIN immunofluorescence staining. 10 ng/ml TGF-β or TNF-α (novoprotein, CK33 or GMP-C008) was respectively used to evaluate the VEGF-C/D expression in mouse or human fibroblasts.

Small interfering RNA (siRNA) Transfection

For gene-silencing studies, siRNA targeting BACE2 and its corresponding negative controls were supplied by GenePharma. The sequences for the siRNA targeting BACE2 was 5'-GACACUGGAAGCAGUAACUTT-3' (sense) and 5'-AGUUACUGCUUCCAGUGUCTT-3' (antisense). Transfections were carried out using Lipofectamine RNAiMAX transfection reagent (Thermo Fisher, 13778150), according to manufacturer's instruction.

Cell Counting Kit-8 (CCK-8) Assay

The proliferation of HuLECs was assessed by CCK-8 assay (Beyotime). 2×10^3 HuLECs were seeded in a 96-well plate with EGM-2 medium. After 6 hours of incubation, non-adherent cells were removed by PBS washing. Subsequently, HuLECs were stimulated with 5% sham or 5/6-Nx mouse serum, with or without 100ng/ml VEGF-C_{156S}. At each indicated time point, the culture medium was replaced with CCK-8 solution for 2 hours, which was then transferred to a new 96-well plate to evaluate the absorbance at 450 nm (SpectraMax Absorbance Reader, Molecular Devices).

In Vitro Scratch and Transwell Assay

To investigate cell migration, HuLECs were treated with mitomycin C (merck 50-07-7, 5ug/ml) to disentangle cell proliferation. Conditional medium from human or mouse fibroblasts were used to affect the migration. For scratch assay, HuLECs were seeded as a monolayer in a 6-well plate until full confluence was achieved. A sterile pipette tip was used to create a straight scratch across the monolayer, simulating a wound. High-resolution images of the scratch were captured after 24 hours using a microscope equipped with a digital camera. To quantify wound closure, the change in scratch width over time was measured using image analysis software. For Transwell assays, 8.0-μm pore membrane filters (Corning, 3422) were inserted. HuLECs (1×10^5 cells/ml, 100 uL) were seeded into the upper chamber of the transwell. The bottom chamber contained 500 μL basic culture medium or conditional medium. After 12 hours incubation, the migrated cells on the lower surface of the transwell filter were fixed in 4% PFA for 10 min and then stained with 1% crystal violet (Beyotime, C0121) for 15 min. High-resolution images of the scratch were captured after 12 hours using a microscope equipped with a digital camera.

Chemotaxis Assay

Chemotaxis assays were conducted by the collagen-coated μ-slides

Chemotaxis kit (80326, Ibidi). HuLECs were seeded into observation chambers at a density of 1×10^6 cells/ml with adherence for 4 hours. Following a washing step, HuLECs were maintained in a serum-free medium. EGM-2 or conditional medium from cephalic vein or AVF fibroblasts were added into left or right chamber. HuLECs migration was monitored over a 24-hour period using a Nikon A1R confocal microscope, with cell track sketching using the “manual tracking” function in ImageJ software.

EdU Assay

For EdU assay, BeyoClick™ EdU Cell Proliferation Kit with Alexa Fluor 488 was used. HuLECs were fixed using 4% paraformaldehyde and stained with DAPI after incubation with 50 mM EdU solution for 2 hours. The EdU labeled cells were photographed under fluorescence microscopy (Olympus).

Supplement Figure & Figure Legend

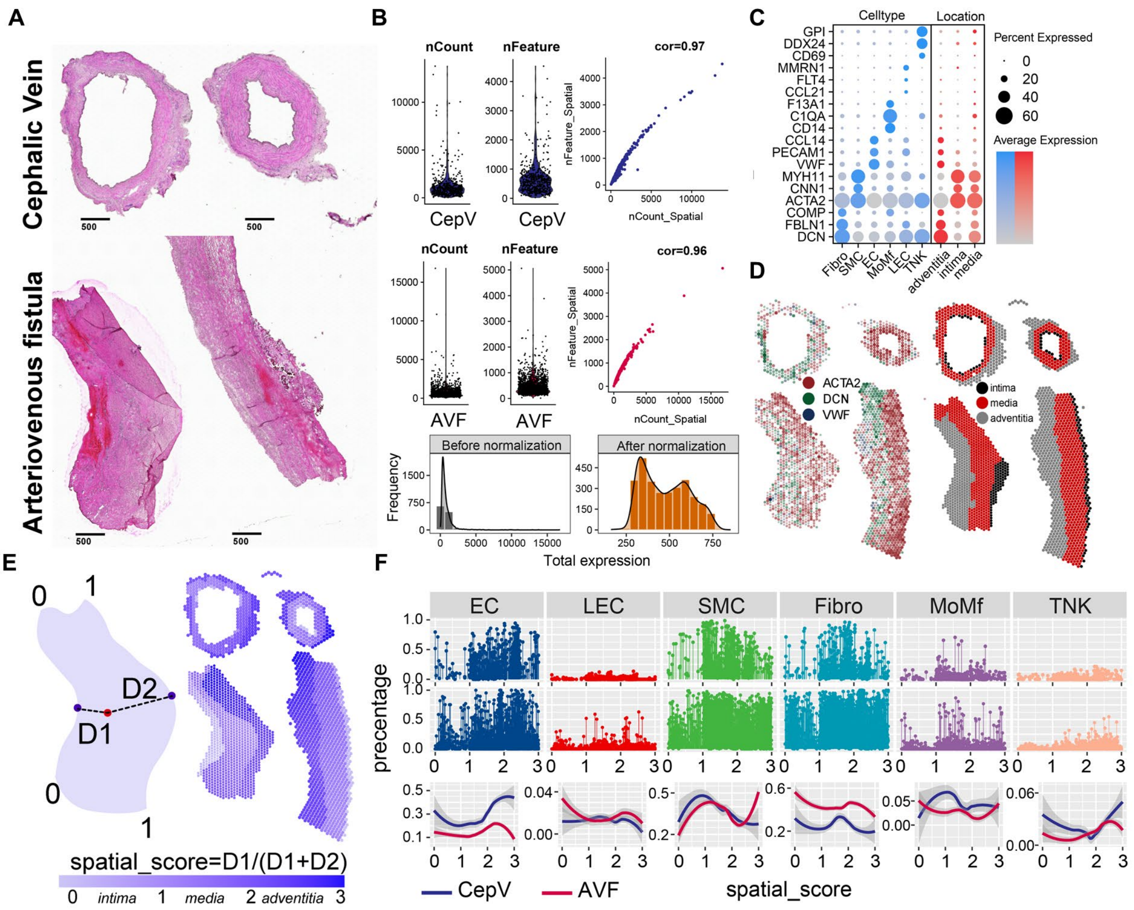

Figure S1. Spatial Transcriptomics of human cephalic veins and arteriovenous fistulas (AVFs). A, H&E staining for human cephalic veins and AVF sections. **B**, Violin plot showing nFeature (total genes) and nCount (total unique molecular identifier, UMI), with Pearson correlation analyses between nFeature and nCount in cephalic veins and AVFs; After using Harmony to merge data from all sections, histogram showing nCount before and after normalization. **C**, Bubble plot showing representative genes expressed by different celltypes or venous layers. **D**, Dot matrix plot showing expression of representative genes in each spot within different venous layers (intima, n=181 spots; media, n=1217 spots; adventitia, n=926 spots). **E**, Schematic diagram of measuring the distances to the nearest points at both edges (D1, lumen; D2, adventitia) to calculate the "spatial score", with dot plot showing spatial distribution of "spatial score": intima (0-1), media (1-2), adventitia (2-3). **F**, Lollipop plot showing celltype percentage in each spot, with line plot displaying

fitting curve of celltype percentages according to the “spatial score”. Scale bars, 500 μm in (**A**).

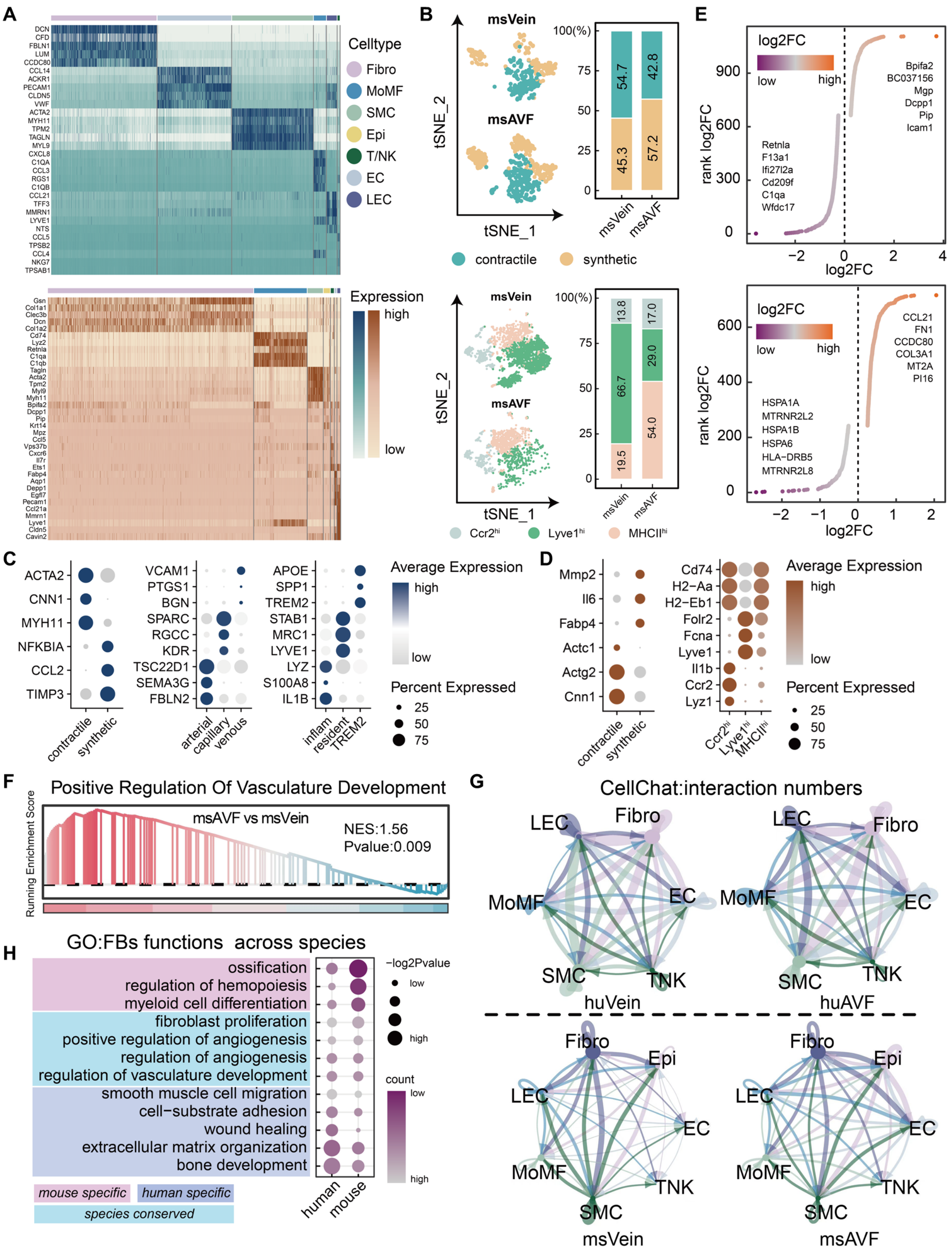

Figure S2. Single-cell RNA Sequencing (scRNA-seq) of control veins and venous segments of AVFs in human and mouse. Similar with **Figure. 2**, we used Harmony to merge scRNA-seq data from control veins and venous segments of AVFs in human or mouse. **A**, Heatmap plot showing expression of top differential genes in each cell cluster: human (top), mouse (bottom). **B**, t-distributed stochastic neighbor embedding (tSNE) plot showing the subclusters of smooth muscle cells (SMCs) and macrophages (Macs) in mouse scRNA-seq data (vein_SMC, n=333; AVF_SMC, n=477; Vein_Mac, n=3000; AVF_Mac, n=1429), with bar plot showing the celltype proportions. **C-D**, Dot plot showing the expression of representative subcluster genes for SMCs, endothelial cells (ECs), or Macs in human (**C**) or mouse (**D**). **E**, Dot plot showing top differentially expressed genes (DEGs) between AVFs and veins from human (top) or mouse (bottom). **F**, Gene Set Enrichment Analysis (GSEA) analysis shows a stronger vasculature regulation function in mouse fibroblasts (FBs) from AVFs. **G**, Circle plot showing interaction numbers among all cell types in control veins and venous segments of AVFs from human or mouse. **H**, Compared with FBs from control veins, bubble plot showing conservative functions enriched with upregulated genes in FBs from human or mouse AVFs. ms, mouse; hu, human; Mo/MF, monocyte/Macrophage; Epi, epithelial cell; and LEC, lymphatic endothelial cell.

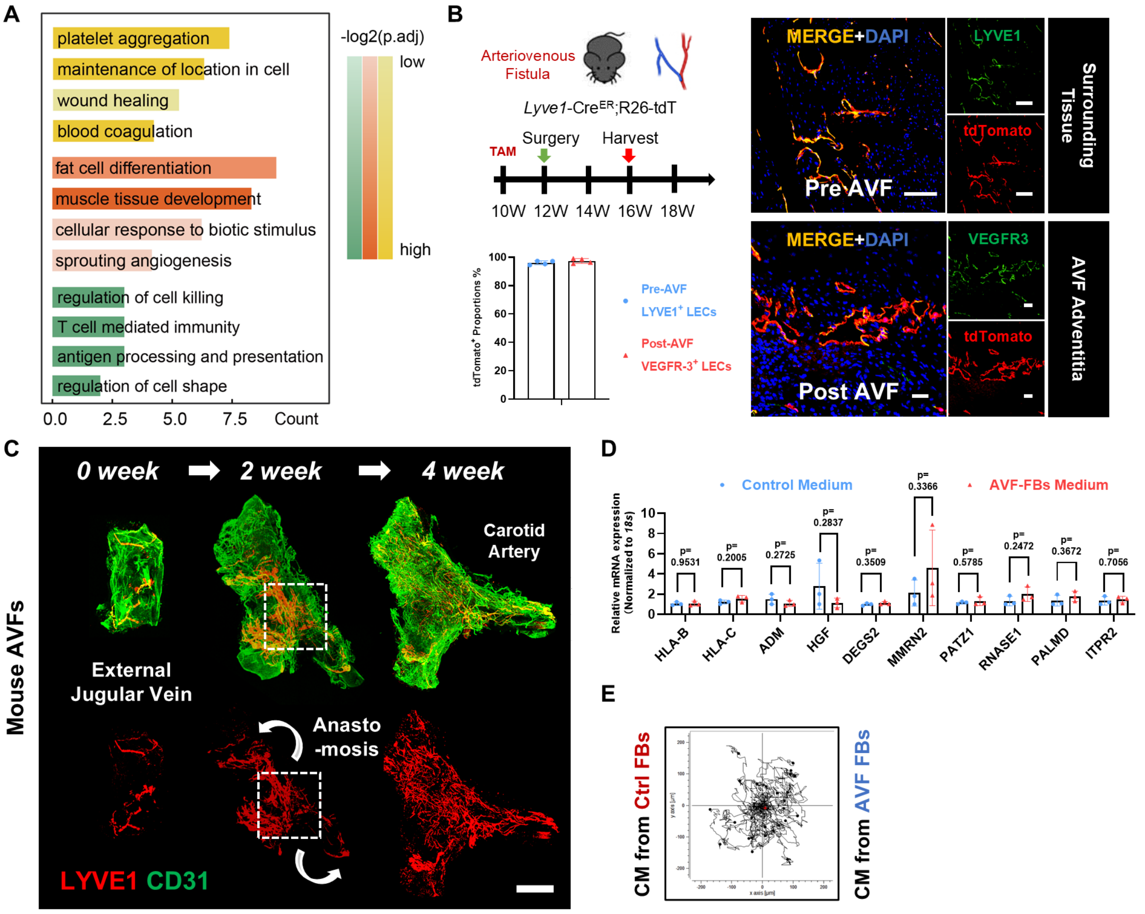

Figure S3. Cellular origin and growth pattern of lymphatic network in mouse AVFs. A, Bar plot showing top differential functions enriched in each mouse LEC subcluster, related to **Figure. 3B**. **B**, Lineage tracing strategy with representative immunostaining for tdTomato (tdT, red) and LYVE1/VEGFR-3 (green) in surrounding tissues (pre AVF) or AVFs (4 weeks post fistulation) from *Lyve1*-Cre^{ER};R26-tdT mice, with corresponding quantifications of tdT^{+} LEC proportion (n=4). **C**, Representative whole-mount staining for LYVE1 (red) and CD31 (green) of mouse AVFs at different time points; Anastomotic sites were shown in white dotted square. **D**, Quantitative PCR analyses of *HLA-B*, *HLA-C*, *ADM*, *HGF*, *DEGS2*, *MMRN2*, *PATZ1*, *RNASE1*, *PALMD*, and *ITPR2* in HuLECs treated with cultured medium from control or AVF FBs (n=3, unpaired two-tailed Student's t-test or Mann-Whitney U test). **E**, Chemotaxis assay of HuLECs in response to supernatants from control (left chamber) or AVF (right chanmer) FBs. Data are mean ± SD. Scale bars, 210 μm in (**B**) and 1000 μm in (**C**). P values of each comparison were specified in the graph.

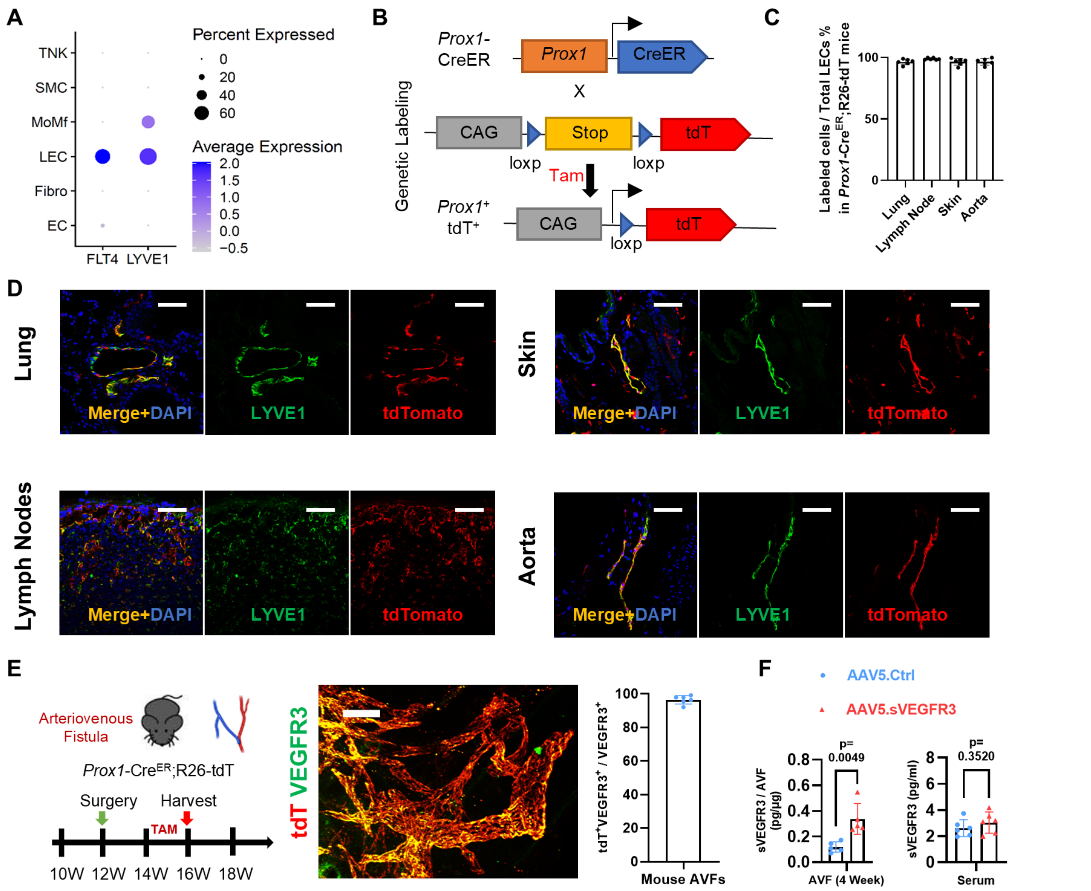

Figure S4. Identifications of VEGFR3$^+$ lymphatic endothelial cells (LECs) in mouse AVFs. A, Dot plot showing the *LYVE1* and *FLT4* expression across the major cell types in AVFs. **B-D**, Schematic diagram illustrating the construction strategy of *Prox1*-Cre^{ER};R26-tdT mice (**B**), with corresponding quantifications of tdT$^+$ proportions in LECs (n=6) (**C**) and representative immunostaining for tdT (red) and LYVE1 (green) in multiple organ sections (lung, lymph nodes, skin, and aorta) (**D**). **E**, Representative immunostaining for tdT (red) and VEGFR3 (green) in AVFs from *Prox1*-Cre^{ER};R26-tdT mice, with corresponding quantifications of VEGFR3$^+$ proportion in tdT$^+$ LECs (n=6). **F**, Quantifications of the sVEGFR3 in serum or AVFs from WT mice, with AAV5.Ctrl (Control) or AAV5.sVEGFR3 infection (n=6, unpaired two-tailed Student's t-test). Data are mean ± SD. Scale bars, 210 μm in (**D**) and 100 μm in (**E**). P values of each comparison were specified in the graph.

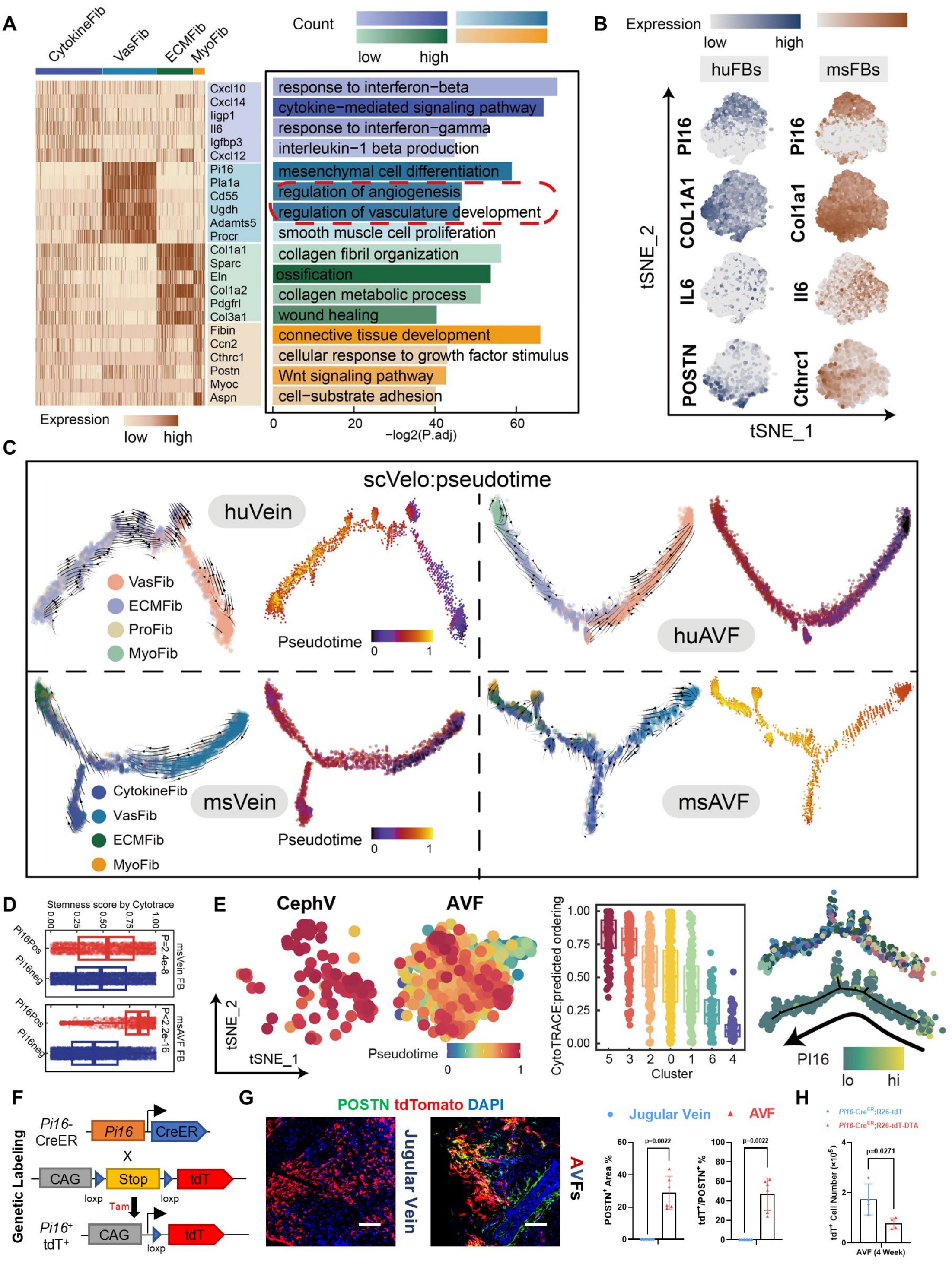

Figure S5. The progenitor-like property of Pi16^{+} FBs in AVFs. A, Heatmap plot showing the top DEGs in each mouse FB subcluster, with bar plot respectively displaying the enriched biological functions (related to Figure. 4A). **B**, Feature plot showing the representative gene expression (*PI16*, *COL1A1*, *IL6*, and *POSTN*) in each fibroblast subcluster. **C**, Streamline diagrams showing the direction of fibroblast differentiation, with DDRTree

algorithm for dimension reduction. **D**, Box plot comparing the score of differentiation potential for mouse Pi16$^+$ or Pi16$^-$ fibroblasts calculated by "CytoTRACE"; Unpaired two-tailed Student's t-test was used. **E**, tSNE plot showing the score of differentiation potential for each spot calculated by "CytoTRACE", with box plot showing the scores of each spot clusters in descending order (related to **Figure. 4D**); Pseudo-time trajectory of each spot with the DDRTree algorithm for dimension reduction according to the *Pi16* expression. **F**, Schematic diagram illustrating the construction strategy of *Pi16*-Cre^{ER};R26-tdT mice. **G**, Representative immunostaining for tdT (red) and POSTN (green) in JVs (pre AVF) or venous segment of AVFs (4 weeks post fistulation) from *Pi16*-Cre^{ER};R26-tdT mice, with corresponding quantifications of POSTN$^+$ and tdT$^+$/POSTN$^+$ proportion (n=6, unpaired Mann-Whitney U test). **H**, Quantifications of tdT$^+$ cell numbers in AVFs from tamoxifen (Tam)-treated *Pi16*-Cre^{ER};R26-tdT or *Pi16*-Cre^{ER};R26-tdT-DTA mice (n=4, unpaired two-tailed Student's t-test). Data are mean ± SD. Scale bars, 210 μm in (**G**). P values of each comparison were specified in the graph.

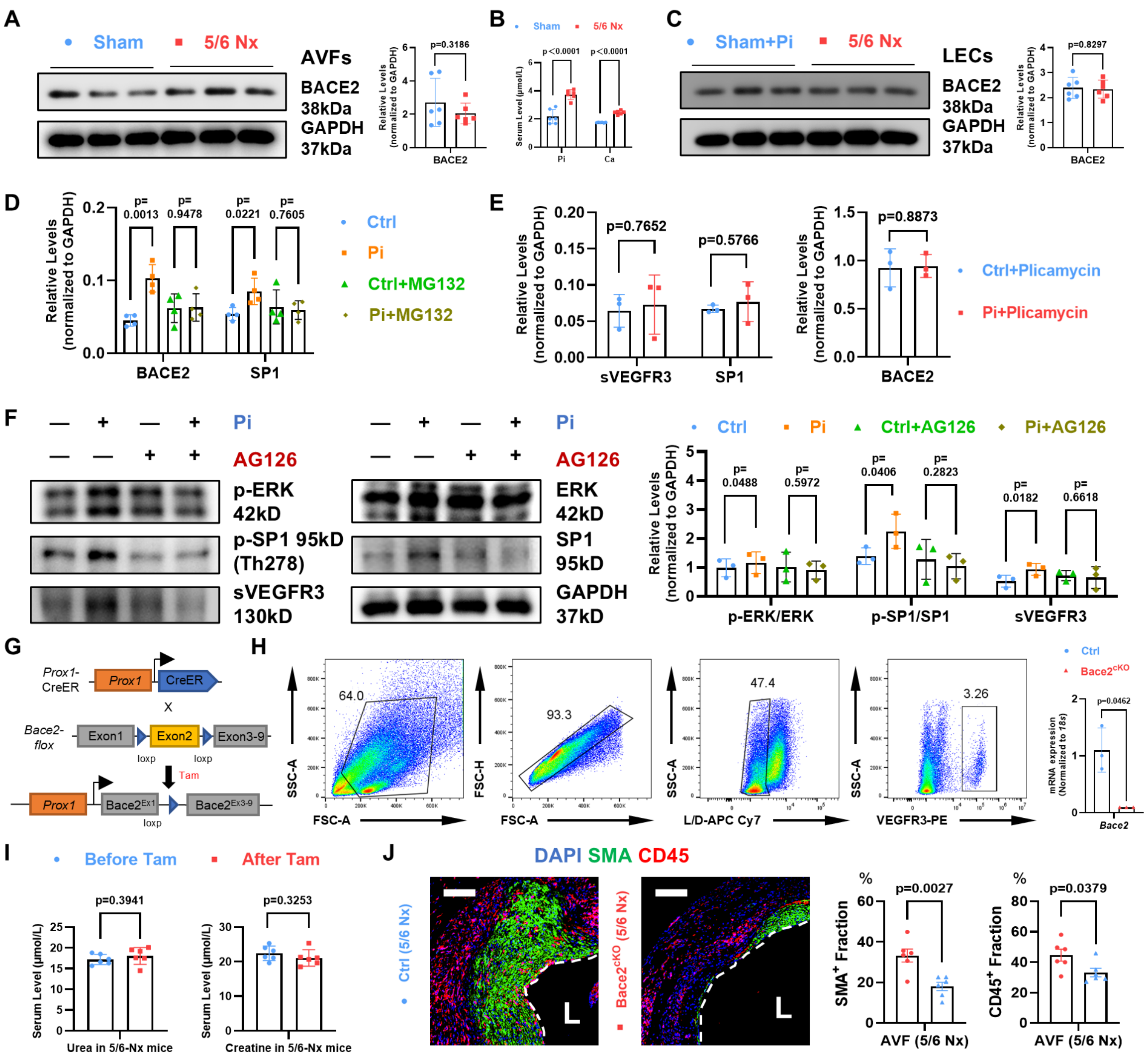

Figure S6. Phosphate (Pi) induced p-ERK/SP1 phosphorylation at Thr278 promotes BACE2 transcription in LECs with high phosphate. A, Western Blot analyses of BACE2 in AVFs from WT or 5/6-Nx mice; Quantifications shown on the right (n=6, unpaired two-tailed Student's t-test). **B**, Serum levels of Pi and calcium (Ca) in Sham or 5/6-Nx mice (n=6, unpaired two-tailed Student's t-test). **C**, Western Blot analyses of BACE2 in HuLECs, treated with Sham serum+Pi (Pi 4μmol/L) or 5/6-Nx serum (Pi 4μmol/L); Quantifications shown on the right (n=6, unpaired two-tailed Student's t-test). **D**, Quantifications of BACE2 and SP1 levels in Ctrl or Pi-treating HuLECs, added with or without MG132 (n=4, , unpaired two-tailed Student's t-test); Related to Figure. 7J. **E**, Quantifications of BACE2 changes in HuLECs in response to Pi stimulation, with or without SP1 inhibitor, Plicamycin, treatment. (n=3,

unpaired two-tailed Student's t-test); Related to Figure 7K. **F**, Western Blot analyses of p-ERK/ERK, p-SP1(Ty278)/SP1, and sVEGFR3 levels in Ctrl or Pi-treating HuLECs, added with or without AG126; Quantifications shown on the right (n=3, unpaired two-tailed Student's t-test). **G**, Schematic diagram illustrating the construction strategy of *Prox1*-Cre^{ER};*Bace2*$^{fl/fl}$ mice. **H**, Quantitative PCR analyses of *Bace2* expression in $VEGFR3^{+}$ LECs isolated from Tam-treated *Prox1*-Cre^{ER} (Control) or *Prox1*-Cre^{ER};*Bace2*$^{fl/fl}$ ($BACE2^{cKO}$) mice (n=3, unpaired two-tailed Student's t-test). **I**, Evaluation of serum creatine or urea in 5/6-Nx mice before and after Tam induction (n=6, unpaired two-tailed Student's t-test). **J**, Representative immunostaining for CD45 (red) and SMA (green) in AVFs from Control or $BACE2^{cKO}$ 5/6-Nx mice, with quantifications of SMA^{+} or $CD45^{+}$ fractions (n=6, unpaired two-tailed Student's t-test). Data are mean ± SD. Scale bars, 210 μm in (**J**). P values of each comparison were specified in the graph. SMA, smooth muscle antigen; and Ctrl, control.

References:

1. Visuri MT, Honkonen KM, Hartiala P, Tervala TV, Halonen PJ, Junkkari H, Knuutinen N, Yla-Herttuala S, Alitalo KK, Saarikko AM. VEGF-C and VEGF-C156S in the pro-lymphangiogenic growth factor therapy of lymphedema: a large animal study. *Angiogenesis*. 2015;18:313-326. doi: 10.1007/s10456-015-9469-2
2. Houssari M, Dumesnil A, Tardif V, Kivelä R, Pizzinat N, Boukhalfa I, Godefroy D, Schapman D, Hemanthakumar KA, Bizou M, et al. Lymphatic and Immune Cell Cross-Talk Regulates Cardiac Recovery After Experimental Myocardial Infarction. *Arteriosclerosis, Thrombosis, and Vascular Biology*. 2020;40:1722-1737. doi: 10.1161/atvbaha.120.314370
3. Chen K, Mou R, Zhu P, Xu X, Wang H, Jiang L, Hu Y, Hu X, Ma L, Xiao Q, et al. The Effect of Lymphangiogenesis in Transplant Arteriosclerosis. *Circulation*. 2023;147:482-497. doi: 10.1161/CIRCULATIONAHA.122.060799

Supplementary *Table S1.*

Number	Sex	Age	BP	Scr	BUN	GFR	TP	SG	Smoke
1	Male	31	117/82	87	5.27	99	60.3	1.017	Yes
2	Female	58	158/95	426	33.09	10	72.8	1.017	No
3	Female	54	165/69	781	19.2	4.6	71.7	1.005	No
4	Male	33	132/95	100	3.51	86	71.2	1.022	No
5	Male	28	145/93	992	18.82	5.29	68.3	1.015	No
6	Male	39	133/88	162	10.17	44.4	63.4	1.015	Yes
7	Male	55	142/95	1600	29.71	2.6	68.9	1.024	Yes
8	Male	48	144/75	158	11.01	43.6	68.3	1.015	No
9	Female	37	149/92	875	18.41	4.58	76.6	1.008	No
10	Female	64	155/76	284	5.14	14.6	70.8	1.012	No
11	Male	33	148/110	803	31.08	6.6	59.8	1.01	No
12	Female	59	146/82	79	5.42	69.83	66.8	1.015	No
13	Male	50	108/57	135	7.88	51.3	66.7	1.016	Yes
14	Female	75	151/66	753	22.64	3.97	57.9	1.008	No
15	Male	85	159/74	543	30.39	7.56	65.3	1.016	Yes
16	Female	64	126/79	611	20.33	5.89	38.7	1.004	No
17	Male	47	139/78	750	20.98	6.7	71.3	1.078	Yes
18	Male	34	126/78	118	6.57	68.5	71.2	1.012	No
19	Male	71	187/89	173	7.07	33.48	54.3	1.014	Yes
20	Male	79	150/65	497	27.3	8.9	56.2	1.005	Yes
21	Male	83	110/75	883	28.64	4.3	61.1	1.014	Yes
22	Male	68	111/68	353	5.3	15	56.3	1.011	Yes
23	Female	46	128/85	564	35.01	7	65.1	1.009	No
24	Female	74	170/92	426	38.33	8	61	1.013	No
25	Male	84	149/68	599	15.25	7	55.4	1.012	Yes
26	Female	60	133/77	91	6.98	60	70.3	1.012	No
27	Male	36	143/96	138	10.98	57	61.8	1.025	Yes

Supplementary *Table S2*.

Gene	Sequence	Comments
Lyve1-Cre^{ER}	AGTTGGGGTTCTGGGAGGGTCAT<br>GAGGCCAGAGGGTAGAGTCCAGA	Genotyping
Pi16-Cre^{ER}	GGATTCTGCTCTGGTCCAGAC<br>CATGTCCATCAGGTTCTTGCGAAC<br>GGATTCTGCTCTGGTCCAGAC<br>CACGGTACTGGTTGTGAAGATCC	Genotyping
Prox1-Cre^{ER}	CCATAAATCCCAGAGCCTATGC<br>TTCCGGTTATTCAACTTGCACC<br>GCATGAAGTGCAAGAACGTGG<br>AGCCAGAAGTCAGATGCTCAAG	Genotyping
R26-tdTomato	CTCTGCTGCCTCCTGGCTTCT<br>CGAGGCGGATCACAAGCAATA<br>TCAATGGGCGGGGGTCGTT	Genotyping
R26-DTA	GCGAAGAGTTTGTCCTCAACC<br>AAAGTCGCTCTGAGTTGTTAT<br>GGAGCGGGAGAAATGGATATG	Genotyping
Vegfc-flox	TTCGCACACATCATCACATAGAGTG<br>GCTCTGGCTTTGATTCCTAACACTAAG	Genotyping
Vegfd-flox	GGGAGAGTGGGAAACTCCAATAGG<br>CAGCCACAGAAAAGCTATAGACTCTCC<br>TCTGAGGCGGAAAGAACCAG<br>CTGCAGACTTTGCAAGCGGAAAC	Genotyping
Bace2-flox	CTTGTGCATCTGGGCTTGAGAACTT<br>CCTGGGAGATACCAGTTTCTGTCTTC<br>TCTGAGGCGGAAAGAACCAG<br>CTGTACTGAAGTCCAGAGGCAGTGA	Genotyping
18S	CGGACAGGATTGACAGATTG<br>CAAATCGCTCCACCAACTAA	qPCR
b-Actin	CCCTGGAGAAGAGCTACGAG<br>CGTACAGGTCTTTGCGGATG	qPCR
HLA-B	CAGTTCGTGAGGTTCGACAG<br>CAGCCGTACATGCTCTGGA	qPCR
HLA-C	CGGTTCTAAAGTCCCCAGTCA<br>CCACGTAGCCCACTGAGATG	qPCR
ADM	ATGAAGCTGGTTTCCGTCG<br>GACATCCGCAGTTCCCTCTT	qPCR
HGF	GCTATCGGGGTAAAGACCTACA<br>CGTAGCGTACCTCTGGATTGC	qPCR
DEGS2	CGGCGCAAGGAGATACTGG<br>GTTGTGCGAGATGTCGTGGA	qPCR

**Continued Supplementary *Table S3***		
MMRN2	AGGACCCCGTTGGACGTAA<br>TTCTGCTTGACCTGGTACACT	qPCR
PATZ1	GATGCACACTATCAGCTCCAAG<br>CGATAACCGACCTCATCAGCA	qPCR
RNASE1	ACTGTAACCAAATGATGAGGCG<br>GTACCTGGAGCCGTTTGTCA	qPCR
PALMD	TGATTTTCATGGGGTATCAGCAG<br>GGGTGGTAGGACGGTTTCT	qPCR
ITPR2	CACCTTGGGGTTAGTGGATGA<br>CTCGGTGTGGTTCCCTTGT	qPCR
SP1	GGAAGTGGAGGCAACATCAT<br>TGAGAGCTGGGAGTCAAGGT	qPCR
BACE2	GGAGATGCTGATCGGGACC<br>AGTACGTGTCTATGTAGGAGTGC	qPCR
Vegfc	GTTACCTCAGCAAGACGTTGT<br>AGGAAGTGTGATTGGCAAAACT	qPCR
Vegfd	TTGAGCGATCATCCCGGTC<br>GCGTGAGTCCATACTGGCAAG	qPCR
Pi16	GGGGCCACAACAAAGAACG<br>CACATCTGGTTCGGATCGCA	qPCR
